# Deciphering the efficacy of staphyloxanthin-encapsulated niosomal nanovesicles to attenuate biofilm formation, quorum sensing, and meropenem persistence in *Acinetobacter baumannii*

**DOI:** 10.1186/s12866-025-04507-1

**Published:** 2025-12-13

**Authors:** Ahmed M. Nosair, Ahmed A. Abdelaziz, Amal M. Abo-Kamar, Lamiaa A. Al-Madboly, Mahmoud H. Farghali

**Affiliations:** Department of Microbiology and Immunology, Faculty of Pharmacy, Tanta University, Tanta, 31527 Egypt

**Keywords:** Acinetobacter baumannii, Staphyloxanthin, Niosomes, Biofilm, Quorum sensing

## Abstract

**Background:**

*Acinetobacter baumannii* is the primary cause of persistent opportunistic infections in healthcare settings, recognized as a global priority due to its resistance to antibiotic therapy. Quorum sensing and biofilm formation are the key factors driving the pathogenesis and drug resistance of *A. baumannii*. Nanostructures demonstrated encouraging promise in enhancing the therapeutic efficacy and overcoming treatment failure. Therefore, the efficacy of staphyloxanthin (STX)-encapsulated niosomes was evaluated both in vitro and in vivo.

**Results:**

The formulated niosomal nanovesicles displayed a spherical shape at the nanoscale (177.8 nm), featuring a slow-release rate (39.6%) and appropriate entrapment efficiency (92.7%). Our results demonstrated that STX exhibited strong antibacterial activity, with MIC values up to 16 µg/mL against multidrug-resistant isolates (*n* = 24). The in vitro findings revealed that the encapsulation of STX within niosomal nanovesicles demonstrated superior therapeutic efficacy compared to the free solution. This improvement was reflected by a significant reduction in biofilm formation (68–88%), motility (66.66–94.45%), and siderophore production (48.75–79.5%), as well as marked disruption of the mature biofilm by 82%. The anti-quorum sensing activity of STX was further confirmed the attenuation of biofilm and virulence, as evidenced by downregulation of *abaI* expression (1.42-fold reduction) and molecular docking simulations. It is noteworthy that the biological findings revealed a significant eradication of meropenem-induced persister cells after the addition of niosomal dispersion. The preclinical investigations prove the efficacy of STX in improving survival rates through reducing the bacterial burden (2-fold reduction) and lethal inflammatory consequences in a mouse model of pneumonia.

**Conclusion:**

our results suggested that STX may serve as a promising alternative for combating *A. baumannii* biofilms and persister cells.

**Supplementary Information:**

The online version contains supplementary material available at 10.1186/s12866-025-04507-1.

## Background

*Acinetobacter baumannii* is an opportunistic human pathogen that predominantly leads to nosocomial infections in individuals with compromised immunity [1]. It is commonly associated with diseases such as ventilator-associated pneumonia, wound infections, meningitis, endocarditis, bacteraemia, as well as infections of the respiratory and urinary tracts. Additionally, *A. baumannii* is one of the most frequently found infections in intensive care neonatal and burn units [1]. The Infectious Diseases Society of America (IDSA) has classified it as a pathogen among “ESKAPE” bacteria owing to its resistance to standard antibiotics [2]. The World Health Organization (WHO) report categorizes this issue as critical within the hierarchy of multidrug-resistant (MDR) bacteria, posing an extreme threat to human health. *A. baumannii* resistance to multiple antibiotics is experiencing a significant increase on a global scale [3]. The development of drug resistance occurs through a variety of mechanisms, involving efflux pumps, secretion systems, alterations to the outer membrane, biofilm formation, and quorum sensing [4]. The two-component AbaI/AbaR system, a homologue of the LuxI/LuxR system seen in other Gram-negative bacteria, comprises up *A. baumannii*’s quorum sensing system. The two-component regulatory system initiates a series of reactions that subsequently modify the expression of various genes regulated by quorum sensing [4]. Antibiotic resistance and the pathogenesis of *A. baumannii* are primarily driven by biofilm formation, a significant factor among the diverse mechanisms of virulence [5].

Biofilms constitute a bacterial community embedded within a matrix of extracellular polysaccharides, proteins, and DNA, adhering to biotic and abiotic surfaces. These structures augment the capacity of bacteria to induce infectious diseases, adhere tenaciously to biological surfaces, circumvent the host’s innate immune defenses, and foster antibiotic resistance [6]. Additionally, patients with prosthetic medical equipment are particularly vulnerable to the serious health issues associated with chronic biofilms [7]. In addition to its multi-drug resistance, *A. baumannii* demonstrates multidrug tolerance, a phenomenon facilitated by persister cells that withstand high concentrations of antibiotics and subsequently contribute to the persistence of chronic infections [8]. This situation underscores the urgent necessity for the advancement of novel antimicrobial treatments.

Staphyloxanthin (STX) is a prominent pigment produced from Staphylococcus aureus and belongs to a particular class of apocarotenoid triterpenoid pigments [9]. The oxidative cleavage of C40 isoprenoids yields apocarotenoids in both plants and microbes. While other processes are employed in the non-enzymatic synthesis of apocarotenoids, carotenoid cleavage dioxygenases (CCDs) are crucial in the enzymatic generation [9]. Most of carotenoids’ biological activity is thought to be due to their propensity to produce reactive oxygen species (ROS), which in turn cause oxidative stress, inhibit ion-membrane interactions, and change the cellular permeability through active solute transport [10]. Recently, a plethora of research has explored the carotenoids and their possible applications in the battle against parasitic and bacterial infections [11–13].

A novel approach to enhance the therapeutic effects of a drug involves administration in the form of nanostructures [14]. Nano-carriers, including niosomes, have been extensively utilized to improve the therapeutic efficacy of drugs and facilitate their delivery. Niosomes are bilayered structures that are composed of biocompatible materials, specifically non-ionic surfactants and cholesterol, which function as lipids [15]. These nanovesicles are extremely biocompatible and water-soluble, with the ability to transport both hydrophobic and hydrophilic agents. Preventing chemical deterioration and the adverse effects of unfavorable environmental conditions, this method of drug delivery substantially enhances the physical stability of the pharmaceutical compound [16]. Regarding the aforementioned scenario, this study relies on the evaluation of STX, both free solution and niosomes, in combating *A. baumannii*.

In this study, the antibacterial activity of STX against *A. baumannii* was evaluated both in vitro and in vivo as a drug solution and niosomal dispersion. Furthermore, STX activity was assessed for attenuation of biofilm formation, quorum sensing, virulence, and meropenem persistence.

## Materials and methods

### Materials

The STX pigment was extracted from *Staphylococcus aureus* A2 (accession number PP197164). Our previous research addressed the laboratory extraction, purification, chemical validation, and safety profile of STX on normal Vero cells [17]. Sorbitan monostearate (Span 60), cholesterol, and dialysis membrane (Sigma Chemical Co., St. Louis, MO, USA) were procured from the Pharmaceutical Technology Department of the Faculty of Pharmacy at Tanta University, Egypt.

### Bacterial isolates and susceptibility pattern determination

Thirty *A. baumannii* isolates were retrieved from microbiological laboratories in the Tanta University Hospitals. The isolation of *A. baumannii* was performed using macConkey agar (HiMedia Laboratories Pvt. Limited, Mumbai, India). Following their recovery, the isolated colonies were presumed to be *A. baumannii* using an assortment of standard biochemical techniques and Gram staining analysis [18]. The isolates were subsequently streaked on HiCrome™ agar (HiMedia Laboratories Pvt. Limited, Mumbai, India), and the light purple colonies were picked up for confirmation by matrix-assisted laser desorption/ionization time-of-flight mass spectrometry (MALDI-TOF MS). For subsequent investigations, the bacterial isolates were kept at −80 °C in tryptic soy broth with 40% (v/v) glycerol. The assessment of antimicrobial susceptibility for the isolated *A. baumannii* was performed using the Kirby-Bauer disc diffusion method on Mueller Hinton agar (MHA) plates [18], according to Clinical and Laboratory Standards Institute (CLSI), 2020 guidelines [19]. *A. baumannii* ATCC 19,606 served as a quality control strain.

### Preparation of niosomal formulation encapsulating staphyloxanthin

The composition of the niosomal formulation listed in Table 1 was utilized following the previously reported investigation [20]. The hydration technique, as previously described, was employed to create niosomes [15, 21]. In essence, cholesterol and span 60 (surfactant) were melted on a water bath at 55 °C. A clear liquid was produced by dissolving STX in 5 mL of ethanol and then adding the solution to the melted components while combining. By progressively incorporating 5 mL of water during the mixing process, a homogeneous mixture was achieved. Proniosomal creamy gel was generated through continuous mixing during chilling. To prepare the surfactant nanovesicles, proniosomes were hydrated. This was achieved by employing continuous mixture to develop homogenous niosomal dispersion, with the remaining water being incrementally incorporated. By overnight incubation at room temperature, the niosomal dispersion was permitted to endure swelling and hydration. Bath sonication was employed to reduce the size of the prepared distended vesicles for a duration of 45 min upon cooling with ice crystals. STX-loaded niosomes were generated at a final concentration of 3 mg/mL in triplicate (*n* = 3).

**Table 1:** Composition of Niosomal formulation

Formulation	Amount or volume
Span 60	1.2 g
Cholesterol	0.3 g
Ethanol	3 mL
Staphyloxanthin	0.075 g
Distilled water	Up to 25 mL

### Characterization of the formulated niosomes

The morphology and size of the produced niosomal formulation were evaluated using transmission electron microscopy (TEM; JEOL – JSM1400 PLUS, Tokyo, Japan) [16]. Niosomes were diluted 1:200 in HPLC-filtered distilled water. A single niosomal dispersion droplet dried on a carbon grid. The grid was then stained with saturated uranyl acetate dissolved in 70% ethanol for 5 min and lead citrate for 2 min. The sample was carefully placed in the holder for TEM. Photomicrographs were obtained by carefully calibrating magnification. Moreover, vesicles’ size and zeta potential were ascertained using Zetasizer Nano-ZS (Malvern Instruments, UK) at 25 °C. A appropriate scattering intensity was achieved by diluting 1 mL of the vesicular dispersion (1:200) with HPLC-filtered distilled water before examination [22]. The measurements were carried out in triplicate (*n* = 3).

### Entrapment efficiency and drug content

The niosomes’ encapsulation effectiveness was assessed after the free STX pigment was separated [15, 23]. The nanovesicles were centrifuged for 90 min at 12,000x g and the liquid phase supernatant was carefully separated from the solid residue pellet. Measurements of absorbance at 456 nm determined the supernatant’s unentrapped STX content. According to the equation, the entrapment effectiveness EE% was obtained by subtracting the free STX content from the total STX employed in vesical formulation: $\;\text{EE}\%\;=\;\lbrack(\text{A}\;-\;\text{B})/\text{A}\rbrack\;\times\;100\%$.

A: Initial STX content in vesical formulation. B: unentrapped STX in supernatant.

The loading efficiency (LE) was STX encapsulated per lipid. As indicated in the calculation, this better showed the niosomes’ ability to load STX compared to the formulation’s total lipid:$\text{Actual encapsulated medication}/\text{formulation lipid}\:=\:\text{LE}\;(\text{mg}/\text{g})$.

### In-vitro drug release

STX release from encapsulated nanovesicles was monitored in-vitro using the dialysis membrane technique [15, 23]. The dialysis membrane was a cellulose acetate membrane with a molecular weight cut-off of 10,500 Da (Sigma-Aldrich, Germany). The donor compartment was loaded with 2 mL of niosomes and submerged in 70 mL of ethanol: PBS (50:50) at pH 7.4. The sink condition was maintained by using this receptor fluid [24, 25]. The system ran at 37 °C with 50 rpm agitation. The blank for UV analysis was a 3 mg/mL solution of STX, identical to the release media employed in the experiment. The blank provides a baseline for the UV-visible spectrophotometer, ensuring reliable drug release measurements without interference from the release medium. Spectrophotometry at 456 nm was used to analyze samples taken at various intervals (60, 120, 180, 240, 300, 360, 420, 480, 600, and 1440 min). The rate of STX release over time was used to determine the drug release kinetics. The measurements were carried out in triplicate (*n* = 3).

### Stability of niosomes

Monitoring niosomes over a predetermined period of time in terms of particular properties is one way to verify their stability [23]. In short, the size and EE% of 1 mL of STX-nisomes were assessed. The requirements were monitored for a month at two different temperatures: 4 and 25 °C.

### Determination of Minimum Inhibitory Concentration (MIC)

According to the CLSI 2020 standard methodology [19], the broth micro-dilution test was employed in order to examine the antibacterial activity. Different dilutions of free STX and niosomes containing STX (2–1024 µg/mL) were tested for MIC values using Mueller-Hinton (MH) broth against challenging exponentially grown tested bacteria with final density of 10^6^ CFU/mL. The microplates were scrutinized after an overnight incubation at 37 °C, with the turbidity of the broth serving as an indicator for the growth of the microorganism under investigation. The MIC value was defined at the lowest concentration with no discernible growth. To determine the minimum bactericidal concentration (MBC), 10 µL of clear wells were spot-spotted onto LB agar plates and incubated at 37 ^◦^C for 24 h. MBC was detected with no growth at the lowest concentration [26, 27].

### Biofilm formation

The capacity of MDR *A. baumannii* isolates (*n* = 24) to promote the formation of biofilm was examined using the crystal violet assay as previously described [28, 29]. In essence, 96-well microtitration plates with tryptic soy broth (TSB) media and 1% glucose were used to cultivate the bacterial isolates for 24 h at 37 °C. After discarding the cultures, phosphate-buffered saline (PBS) was used to wash the plates twice. After 15 min of 0.1% crystal violet staining the ensuing biofilm, the leftover stain was washed off with PBS. Using a microtiter reader (SunriseTM, TECAN, Switzerland), the stained-biofilm was solubilized by 33% (vol/vol) glacial acetic acid and measured by absorbance at 595 nm. After biofilm quantification, strong biofilm-producing isolates were selected for further investigation.

### Effect on growth kinetics

After inoculating strong biofilm-producing isolates (*n* = 8) into double-strength LB broth (with an optical density of 600 nm = 0.3), 100 µL was dispensed into each well of a microtiter plate. Subsequently, 100 µL of STX at concentrations of 0, 0.25, 0.5, and 0.75 MIC was aspirated with a micropipette, and the mixture was thoroughly combined. A microplate reader (Sunrise™, TECAN, Switzerland) measured optical densities at 630 nm at intervals of 0, 2, 4, 6, and 12 h. The impact of STX-niosomes on the growth of *A. baumannii* isolate*s* was illustrated by plotting the observed optical densities over time (h) [30].

### Biofilm inhibition assay

The impact of free STX and niosomes on biofilm formation were evaluated as previously described [31]. Briefly, overnight cultures of the tested isolated were diluted to 0.5 McFarland (1.5 × 10^8^ CFU mL^− 1^) and incubated with the treatments (0.5 MIC) for 24 h at 37 °C. Biofilm biomass was quantified using crystal violet staining. Lastly, the percentage of biofilm inhibition was determined by applying the following formula: (%) = [(Control OD_570_nm - Treated OD_570_nm)/Control OD_570_nm] × 100.

### Air–liquid interface biofilm formation assay

To evaluate the impact of STX on ring biofilm formation at the air–liquid interface, *A. baumannii* isolates were grown for 24 h at 37 °C in glass tubes with 2 mL of TSB supplemented with or without STX treatment (0.5 MIC). The tubes were rinsed three times with PBS after incubation and then stained with a 0.4% crystal violet solution [4].

### Assessment of pellicle formation

The methodology for testing pellicle formation was adapted from earlier approaches [28, 32]. In summary, overnight bacterial cultures were diluted 1:100 in 5 ml TSB and incubated in glass tubes at 37 °C for 72 h under static condition. The pellicle material was quantified by introducing 1 ml of ethanol into the tube, extracting the floating pellicles, and resuspending them in PBS. A spectrophotometer (SunriseTM, TECAN, Switzerland) was employed to quantify OD_600_ values.

### Microbial Adhesion to Hydrocarbon assay (MATH)

The MATH test was employed to evaluate the impact of STX on the cell surface hydrophobicity (CSH) of *A. baumannii*, as previously mentioned [33]. Briefly, bacterial isolated were inoculated in TSB without and with treatment (0.5 MIC) and incubated at 37 °C for 24 h. The cells were centrifuged at 12,000 rpm for 15 min. Following three rinses with 1 PBS, the absorbance of the cell solution was measured at 600 nm. Subsequently, 1 mL of toluene was incorporated into the cell suspension and vortexed for 10 min. The solution was allowed to undergo phase separation. Subsequent to the removal of the organic phase, the optical density (OD) of the cell suspension was assessed at 600 nm. The hydrophobicity index was derived from CSH use the formula:

Hydrophobicity index = (1 - OD600 after vortexing/OD600 before vortexing)) x 100.

### Quantification of exopolysaccharide (EPS)

The impact of STX on the production of EPS was previously evaluated [26]. In brief, *A. baumannii* isolates were incubated in LB broth with and without treatment (0.5 MIC). Following a 24-h incubation period at 37 °C, the particles were suspended in PBS and subsequently centrifuged at 8000 × g for 10 min. The supernatant was centrifuged after being combined with an equal volume of ethyl alcohol. Finally, the EPS solution (1 mL) was meticulously combined with cold 5% phenol (1 mL) and concentrated sulphuric acid (5 mL). The optical density of the resultant red color at 490 nm was measured to determine the percentage of reduction in EPS following STX treatment.

### Biofilm disruption assay

The biofilm-eradicating efficacy of STX was evaluated by inducing a biofilm of strong biofilm-producing isolates in a 96-well polystyrene microtiter plate for 48 h. The adhered phase was subsequently rinsed twice with PBS after the planktonic cells were removed. The remaining adhered cells were subsequently treated with 200 µL of tryptic soya broth (TSB) medium alone or treatment (MIC) for 24 h at 37 ^◦^C. Total biomass and biofilm-adhered cell viability were measured. Biofilms were scraped and resuspended in PBS to count colony-forming unit (CFU). Following serial dilution, 100 µL of each dilution was plated on LB agar and incubated at 37 °C for 24 h. Subsequently, the crystal violet staining assay was employed to quantify the total biomass [34]. To verify the eradication activity, STX’s ability to destroy the mature biofilm was further evaluated on glass surfaces. In a 6-well plate, biofilms were formed on coverslips. Following treatment, biofilms were visualized using a compound bright field microscope (Labomed, California, America) at a magnification of ×40 after being stained with crystal violet [35].

### Confocal Laser Scanning Microscopy (CLSM)

The biofilm eradication activity of STX on the established biofilm was visualized under a CLSM [26, 34]. The biofilm was induced into an 8-well µ--Slide (ibidi, Martinsried, Germany) after 48 h. The Live/Dead fluorescent dye (Invitrogen) was used to stain the wells following the treatment. Subsequently, biofilms were examined under CLSM at ×20 magnification (DMi8; Leica Microsystems). Dead cells were labelled with propidium iodide (red fluorescent), while viable cells were labelled with acridine orange (green fluorescent).

### Motility assay

The impact of STX treatments (free and niosomes) on the motility of *A. baumannii isolates* was assessed through swarming and twitching motility assays [4, 28]. To perform the swarming motility, 0.5% MHA plates were prepared with and without STX treatments at concentrations of 0.5 MIC. Subsequently, 5 µL of overnight culture was introduced to the central region of the agar plates and incubated at 37 °C for 72 h. The motility zone was assessed following incubation. To test twitching motility, a sterile toothpick was used to pierce the overnight culture on a 1% MHA plate from the middle to the bottom. The plate was then incubated at 37 °C for 72 h. Plates were rinsed with PBS and stained with 0.4% crystal violet solution after the agar was properly eliminated.

### Siderophore estimation assay

The impact of STX treatments (free and niosomes) on the extent of siderophores secreted by bacterial isolates was assessed by the universal Chrom Azurol S (CAS) assay [36]. In a nutshell, the bacterial cultures were incubated at 37 °C for 24 h in 5 mL of TSB with and without treatment at a concentration of 0.5 MIC. Following incubation, 1 mL of the bacterial cultures was centrifuged at 4900x g for 20 min. In triplicate, 100 µL of the siderophore-containing supernatant was transferred to 96-well microtitration plates, which already contained 100 µL of CAS-hexadecyl trimethyl ammonium bromide (CAS-HDTMA) solution. A blank and positive control were established in wells that contained only fresh medium and 15 mM EDTA, respectively. The plates were incubated for 30 min in the dark. Lastly, the plate reader was employed to measure the absorbance at a wavelength of 630 nm. According to the following equation, the siderophore content is then calculated.

% siderophore units = ((A_r_ - A_s_)/A_r_) x 100.

In this equation, A_r_ represents the reference absorbance (blank), while A_s_ denotes the absorbance of the supernatant.

### Gene expression analysis

The protocol provided by Roche Diagnostic GmbH (Germany) was followed to extract total RNA from bacterial pellets. Using absorbance measurements with samples having a 260/280 nm ratio in the 1.8–2.8 range, the RNA yield and purity were evaluated. Following the directions given by the supplier of the kit (Thermo-Fisher Scientific, Waltham, MA, USA), the cDNA synthesis was carried out. To determine gene transcript levels using the oligonucleotides listed in Table 2, a qRT-PCR was performed using a Rotor-Gene Q apparatus (Qiagen, USA). The 16 S rRNA gene, serving as a housekeeping gene, underwent 30 cycles consisting of denaturation at 94 °C for 30 s, annealing at 60 °C for 30 s, extension at 72 °C for 30 s, followed by a final extension at 72 °C for 5 min. The cycling parameters were established at 94 °C for a duration of 3 min [26]. Expression data were obtained from three independent biological replicates (*n* = 3), each measured in triplicate (technical replicates). 16 S rRNA was validated as a stable housekeeping gene under the tested conditions and used for normalization. Relative expression levels of *abaI* were calculated using the 2^−ΔΔCt^ method.

**Table 2:** Primer sequence involved in real-time PCR

Primer name	Primer sequence	Amplicon size (bp)	Efficiency (%)	References
16 S rRNA	F: 5’-TAGTCCATGCCGTAAACGATGT-3’ R: 5’-TTGAGTTTTAGTCTTGCGACCG-3’	114	108%	[37]
*abaI*	F: 5’-CCACACAACCCTATTTACTCGC-3’ R: 5’- GGCGGTTTTGAAAAATCTACGG − 3’	121	101%	[38]

### Molecular Docking

Molecular docking research were carried out using the Molecular Operating Environment (MOE, 2019.0102). The MMFF94x force field was used to automatically calculate the partial charges once all MOE minimizations were completed up to a gradient of 0.05 kcal mol − 1 Å−1. The protein chains that were going to be used in the molecular docking simulations were prepared for them by removing all residues and adding polar hydrogens using the protonate 3D procedure in MOE with its default parameters. This created protonation states that worked well in the simulations. In order to construct docking poses, the Triangle Matcher placement method and the London-dG scoring function were employed [17].

### Anti-persister assay

The persister assay was conducted as previously outlined, with minor modifications [8, 39]. Biphasic time-kill curves under antibiotic stress were first determined by challenging the isolates A1, A3, A7, and A8 (initial inoculum size of OD600 = 0.5) with meropenem at concentrations of 5X MIC over an 8-h incubation period. For the persister assay, cells were grown to exponential phase (OD600 = 0.5), and 1 mL of the culture was exposed to meropenem (5X MIC) at 37 °C. After incubation with shaking at 185 rpm for 4 h, the samples were washed twice with PBS and subsequently distributed on LB agar using serial dilution. The colony-forming unit (CFU) for surviving persister populations was ascertained. The harvested persister cells were re-grown in LB broth overnight and then treated with 5X meropenem, as mentioned earlier. This cycle was reiterated thrice. To confirm that the surviving colonies were persister cells, one colony was re-inoculated into new LB broth and tested for antibiotic sensitivity to ensure that the MIC was not altered. The impact of STX (both free form and niosomes) on persister cells formation was assessed by applying 5X MIC of STX treatments in late exponential phase bacterial cultures in presence of meropenem (0.5 MIC). After, a 6-h incubation period, the CFU for surviving persister populations was enumerated, and survival rates after treatments were determined [40].

To test the efficacy of STX in accelerating cell death of pre-formed persister cells, STX (5X MIC) was added to *A. baumannii* late exponential phase cells treated with 5X MIC meropenem for 6 h. The survival rate of pre-formed persister cells was examined after STX treatments [39].

### Respiratory infection model

This study employed a total of 24 male Swiss albino mice weighing an average of 25 g, obtained from the Faculty of Pharmacy at Tanta University. We obtained an informed consent from Faculty of Pharmacy at Tanta University to use the animals, and the Research Ethics Committee of the Faculty of Pharmacy at Tanta University (approval code, TP/RE/6/25 p-002) approved this study and the procedures in accordance with ARRIVE guidelines. Mice were given unfettered access to water and a regular pellet diet. In order to produce transitory neutropenia in mice, cyclophosphamide (300 mg/kg body weight) was injected intraperitoneally at days − 4 and − 1 before infection, after a one-week adaption period [41, 42]. Thiopental was administered via intraperitoneal injection at a volume of 0.08–0.1 mL, corresponding to a dosage of 40 mg/kg body weight. After light anesthesia, intranasal administration of *A. baumannii* (final concentration of 10^6^ CFU/mL in sterile PBS) established a respiratory infection. Animals were then categorized into four separate groups. Group I functioned as a negative control: uninfected and untreated. Group II functioned as a positive control, undergoing infection without intervention. Group III: infected and administered STX pigment dissolved in 1% DMSO at 6 h post infection. Group IV: infected and administered the STX niosomal formulation at 6 h post infection. Over the course of 7 days, survival rates, clinical symptoms, and disease progression were monitored. From 0 to 3, the subsequent clinical indications were assessed: Healthy, normal mice at the 0th stage. There was a significant noise response and motility in Stage (1) Weight loss, whiter ears, and moderate noise and motility reaction are all evidence of Stage (2) In the third stage, death and cyanosis occurred, concomitant with minor disturbance response and movement [27].

### Bacteriological and histopathological examination

Lung tissues were aseptically collected for further analysis following the euthanasia (by cervical dislocation under light anesthesia) of three randomly selected animals from each group 48 h post-infection. A tissue homogenizer was employed to weigh and homogenize the right lungs in sterile PBS. Lung homogenates were diluted and subsequently plated onto LB agar to quantify the viable bacterial count. In contrast, 10% neutral buffered formalin was utilized to preserve the excised left lungs. Hematoxylin-eosin (H&E) staining was utilized on the paraffin-embedded tissue [41]. A grading system was implemented to compare the lesions in the untreated and treated groups of mice, taking into consideration the most prominent observable aspects of the lungs [27].

### Statistical analysis

GraphPad Prism version 5 was employed to conduct the statistical analysis. Data were given as mean ± SD, with significance considered at *p* < 0.05. To compare more than two groups, ANOVA test was employed, and pairwise comparisons were made using the Tukey’s post hoc test. Student’s t-test was performed to compare the two groups.

## Results

### Bacterial isolated and antibiotic susceptibility

The isolation of *A. baumannii* strains revealed that the highest frequencies were found in endotracheal fluids (*n* = 9, 30%), sputum (*n* = 8, 27%), and wound samples (*n* = 7, 23.3%). In contrast, urine samples (*n* = 4, 13.3%) and blood samples (*n* = 2, 6.6%) exhibited relatively lower frequencies. The respiratory tract emerges as the predominant site for infection by *A. baumannii*, exhibiting an isolation frequency exceeding 50%, with contributions of 30% from endotracheal fluid and 27% from sputum. A panel of sixteen antibiotics spanning nine distinct categories were employed to assess the antibiotic susceptibility of the identified isolates of *A. baumannii* (Table 3). According to Magiorakos et al. [43], the majority of the isolates exhibited multi-drug resistance (*n* = 24, 80%), with at least one agent demonstrating resistance across at least three distinct categories. It is important to highlight that all isolates exhibit sensitivity to colistin, with the CLSI breakpoint for colistin resistance set at 2 µg/mL.

**Table 3:**
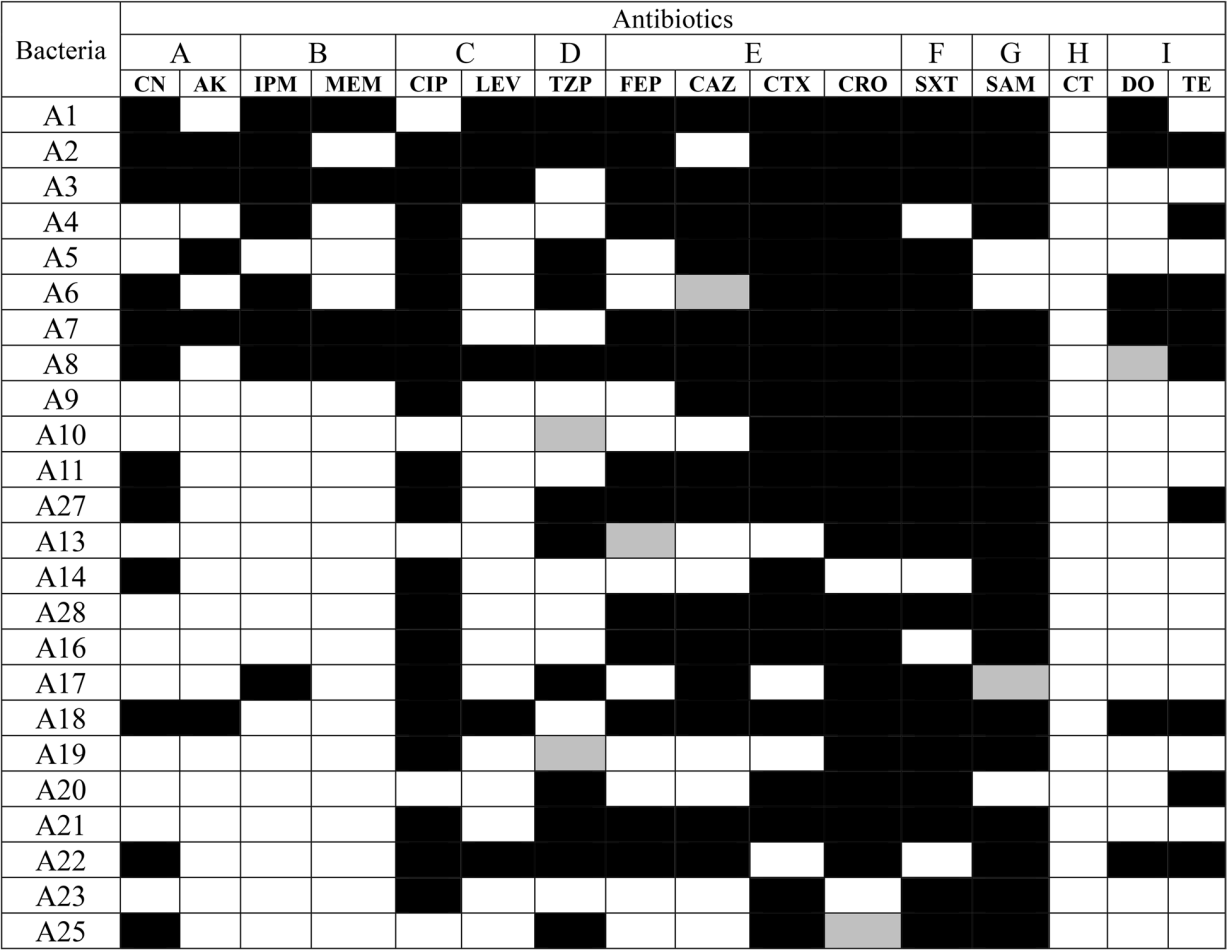
Antibiotic susceptibility profile of *A. baumannii* isolates utilized in the present investigation.Bacteria were categorized as sensitive (white box), intermediate (grey box), or resistant (black box) in accordance with CLSI criteria

*A* Aminoglycosides, *B* Antipseudomonal carbapenems, *C* Antipseudomonal fluoroquinolones, *D* Antipseudomonal penicillins + β-lactamase inhibitors, *E* Extended-spectrum cephalosporins, *F* Folate pathway inhibitors, *G* Penicillins + β-lactamase inhibitors, *H* Polymyxins, *I* Tetracyclines

### Niosomal formulation exhibited acceptable nanoscale morphology, in vitro release, and stability profile

**Fig. 1:**
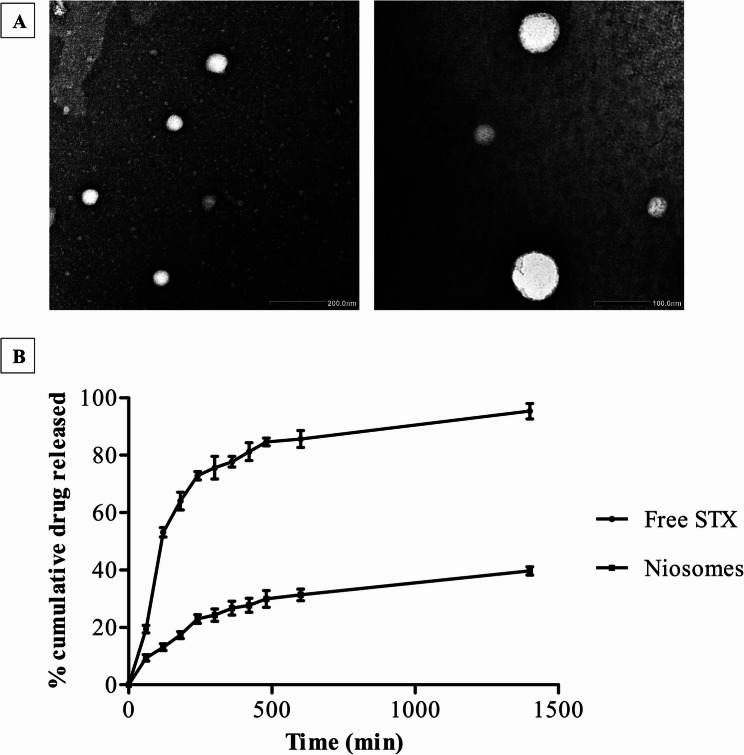
Characterization of the formulated niosomes. (**A**) Representative TEM micrographs of niosomes showing spherical vesicles within the nanoscale range. (**B**) The in-vitro release profile of STX at 37 °C in free form and niosomes (2 mL donor volume; sink conditions). Data was expressed as mean ± SD (*n* = 3)

The evaluation of the morphology, zeta potential, and particle size of the niosomal formulation was conducted using TEM and DLS. Representative TEM micrographs of the formulated niosomes are depicted in Fig. 1A. The micrographs depict spherical particles exhibiting diameters within the nanoscale range. The DLS measurement provided further validation of the vesicle size, confirming that it was within the nanoscale range, with an average size of 177.8 nm (supplementary material Fig. S1). The polydispersity index value recorded at 0.383 suggests that the niosomes produced exhibited a uniform vesicle size. The findings revealed that the zeta potential of the synthesized niosomes is a notable negative value of −35.23 ± 1.26 (supplementary material Fig. S1), highlighting the robustness and stability of the niosomal vesicles. The encapsulation efficiency of STX within niosomal vesicles is documented at 92.7%. The assessment of drug loading involved quantifying the amount of drug encapsulated per gram of lipid. The results demonstrated that 185.4 mg of STX was integrated per gram of lipid in the formulation.

The release profile of STX was observed to be affected by the cholesterol and surfactant content within the niosomes. The findings depicted in Fig. 1B demonstrated that the release of STX encapsulated in niosomal nanovesicles has a slower release than free solution. After 24 h, the niosomal vesicles exhibited a release percentage of 39.6 + 2.51%, in contrast to the free form (95.3 + 4.72%). Over a 30-day period, the stability of niosomes was evaluated under storage conditions of ambient temperature and refrigeration (4 °C). Our study’s findings demonstrated that niosomes kept in a refrigerator at 4 °C exhibited greater stability, with no discernible sedimentation or creaming over the storage period, and the niosomal suspension retained its initial particle size and EE%. On the other hand, the niosomes stored at the temperature of 25 °C showed comparable changes in their initial particle size (836.2 nm; supplementary material Fig. S2) and EE% (85.3%).

### Killing activity and growth Inhibition

Using the broth microdilution method, the antimicrobial activity of STX against isolates of MDR *A. baumannii* was investigated. The observed MIC values of the tested agents exhibited variability contingent upon the examined isolates. The findings presented in Table 4 indicated that the MIC values ranged from 32 to 128 µg/mL, with a significant proportion of the isolates (*n* = 19, 80%) demonstrating a MIC value of 64 µg/mL. The encapsulation of STX within niosomes led to a significant decrease in the MIC values. The boost in efficiency was clearly observable across all isolates, with reductions ranging from 2 to 8 folds. There was no discernible growth when treated bacteria were sub-cultured on LB agar plates, suggesting that the tested agent has a bactericidal mode of action.

**Table 4:** Biofilm-forming capacities and STX susceptibility (free and niosomes) of MDR *A. baumannii* isolates

Bacterial isolates	Biofilm formation	Free STX		STX-niosomes	
		MIC (µg/mL)	MBC (µg/mL)	MIC (µg/mL)	MBC (µg/mL)
*A. baumannii* ATCC 19,606	WBP	32	32	8	8
A1	SBP	64	64	16	16
A2	SBP	64	64	16	16
A3	SBP	64	64	16	16
A4	MBP	64	64	8	8
A5	MBP	64	64	16	16
A6	SBP	64	64	16	16
A7	SBP	64	64	32	32
A8	SBP	64	64	32	32
A9	MBP	64	64	16	16
A10	WBP	64	64	16	16
A11	MBP	64	64	16	16
A27	MBP	128	128	32	32
A13	MBP	32	32	8	8
A14	WBP	32	64	16	16
A28	MBP	32	64	8	8
A16	MBP	64	64	16	16
A17	WBP	64	64	16	16
A18	SBP	64	64	16	16
A19	WBP	64	64	16	16
A20	WBP	64	64	16	16
A21	MBP	64	64	16	16
A22	SBP	128	128	16	32
A23	WBP	64	64	16	16
A25	MBP	64	64	16	16

*SBP* strong-biofilm producer, *MBP* moderate-biofilm producer, *WBP* weak-biofilm producer

**Fig. 2:**
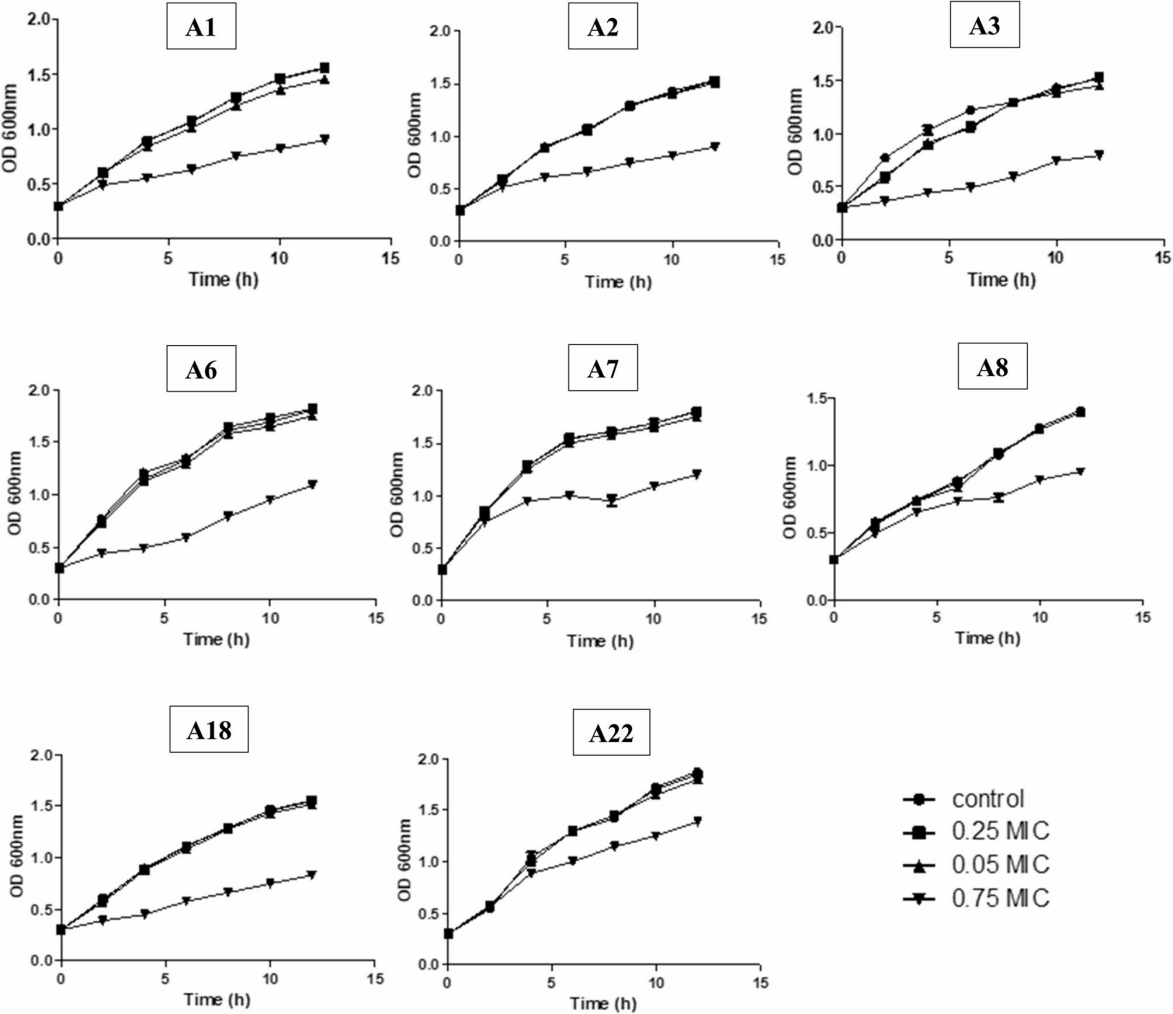
Growth curve of *A. baumannii* isolates (*n* = 8) cultured with and without 0.75, 0.5, and 0.25 MICs of STX at 2-h intervals. The average of three sample replicates produced the results

*A. baumannii* isolates experiencing strong biofilm production capacity (Table 4), as determined by the crystal violet method, were chosen to assess the efficacy of the tested agents against both planktonic cells and biofilm. Firstly, the growth of planktonic cells was challenged with different concentrations (0.25, 0.5, and 0.75 MIC). The dose-dependent growth inhibition was investigated by comparing the spectrophotometric OD values over a 12-h period. The growth curves demonstrate that STX at a concentration of 0.75 MIC substantially impeded the growth of *A. baumannii* planktonic cells under shaking conditions (Fig. 2). Nevertheless, other concentrations exhibited minimal impact on the growth. Consequently, these concentrations may serve as an approach for regulating biofilm formation while preserving the viability of the planktonic cells. These findings indicated that STX’s antibiofilm activity was not a consequence of its killing activity, suggesting that it may be less vulnerable to the emergence of drug resilience.

### Staphyloxanthin impedes microbial adhesion and biofilm formation

**Fig. 3:**
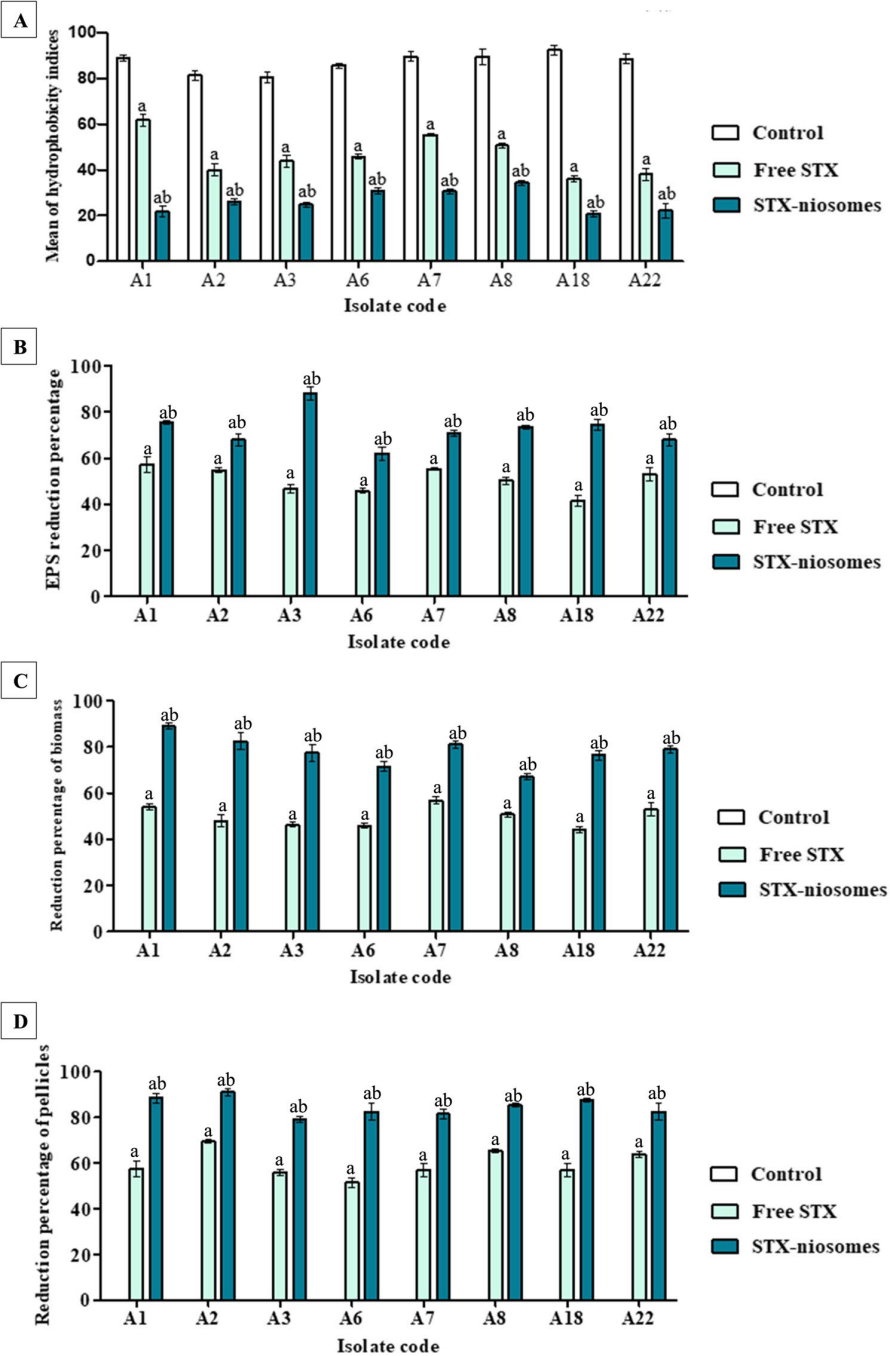
Effect on cellular adhesion and biofilm formation of strong biofilm producing *A. baumannii* isolates. (**A**) Bar chart indicates the reduction percentage of cell surface hydrophobicity after treatment. (**B**) Bar chart indicates the reduction percentage of exopolysaccharide formation after treatment. (**C**) Bar chart indicates the reduction percentage of biofilm biomass after treatment. (**D**) Bar chart indicates the reduction percentage of pellicle formation after treatment. Statistical significance is denoted by the letters, at *P <* 0.05 (one-way ANOVA). Letter a indicates statistical significance compared to control. Letter b indicates statistical significance compared to treatment (free STX). Data expressed as mean ± SD (*n* = 3)

The formation of biofilms is profoundly influenced by cell surface hydrophobicity (CSH), which is a metric that estimates a cell’s affinity for hydrophobic environs. The cell adherence to hydrophobic substratum was evaluated by MATH assay. The findings illustrated in Fig. 3A indicated that the untreated hydrophobic cells exhibit a heightened resemblance for non-polar solvent (toluene). Following treatment, the affinity of cells for the solvent diminished by 29.44–56.87% (*P* < 0.05), with the most notable enhancement occurring in the case of nanovesicles, which exhibited a substantial decrease in hydrophobicity indices of 65.75–76.8% (*P* < 0.05). The impact on exopolysaccharide production, the key part of the biofilm matrix, was assessed using the phenol–sulfuric acid method. The dark red shade of the supernatant and the OD values showed that the untreated cells produced a significant amount of exopolysaccharide after incubation (Fig. 3B; supplementary material S3). Treatment with STX significantly (*P* < 0.05) reduced the production of exopolysaccharide by 43.2–59.5%, with the highest reduction observed in the case of the niosomal formulation (64.9–86.4%).

Taking into the account the overall biofilm biomass, the crystal violet OD values of all examined isolates exhibiting associated biofilms experienced a significant reduction (*P* < 0.05) of 43% to 58% upon treatment with STX solution. Administration of niosomal dispersion significantly (*P* < 0.05) enhanced the efficacy of STX, resulting in an attenuation of the biofilm formation by 68–88% (Fig. 3C; supplementary material S3). Considering the colonization of *A. baumannii* on the upper surfaces of stagnant media and biofilm creation at air-liquid interfaces, the inhibitory effect on pellicle and ring biofilm formation was evaluated. After a span of 24-h incubation, the liquid’s surface commenced to form a fine pellicle, and then a dense and opaque pellicle had completely covered the whole surface of the liquid by the close of the third day (Fig. 3H). Treatment with tested agents resulted in a significant reduction (*P* < 0.05) of pellicle formation by 50–69% (STX) and 78–92% (niosomes), as shown in Fig. 3D. The findings revealed that STX (free and nanovesicles) markedly reduced the ring biofilm formation (crystal violet staining) in all tested *A. baumannii* isolates (supplementary material S3).

### Encapsulation of staphyloxanthin effectively disrupt the mature biofilm

**Fig. 4:**
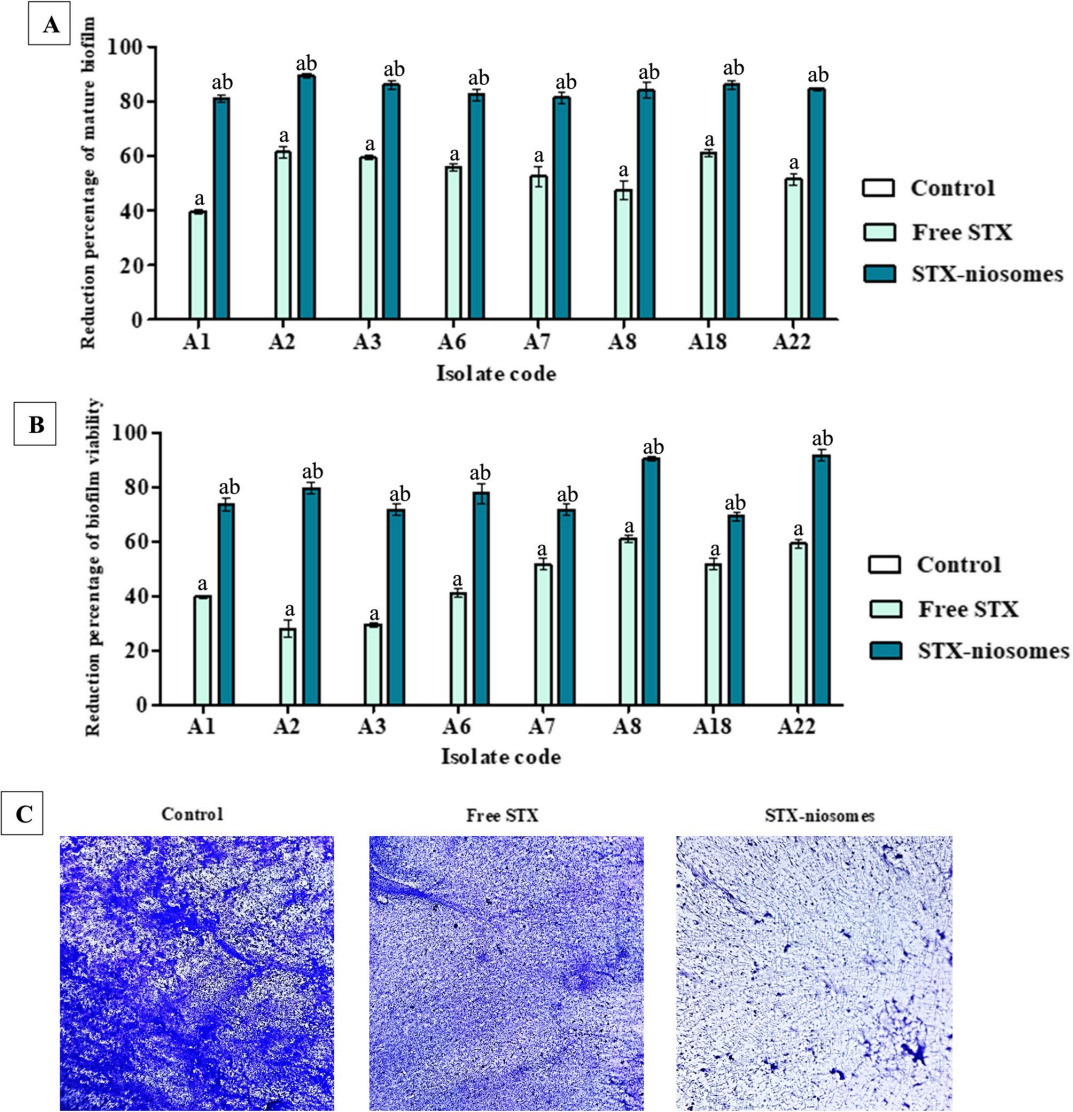
Encapsulation of STX effectively eliminated the 48-h mature biofilms produced by various *A. baumannii* isolates. (**A**) Bar chart indicates the reduction percentage of the total biomass of attached biofilm after treatment. (**B**) Bar chart indicates the reduction percentage of the viability of biofilm-adhered cells after treatment. (**C**) Representative images the preformed biofilm under bright field microscope (×40) with and without treatment. Statistical significance is denoted by the letters, at *P <* 0.05 (one-way ANOVA). Letter a indicates statistical significance compared to control. Letter b indicates statistical significance compared to treatment (free STX). Data expressed as mean ± SD (*n* = 3)

The potential efficacy of free STX and its formulation for biofilm disruption was evaluated against strong biofilm-producing isolates (Table 4). Concerning the total biofilm biomass, STX treatment of 48-h pre-established biofilm significantly decreased the total attached biomass, as indicated by crystal violet OD values, for all tested isolates, with reductions of 51% for the solution and 82% for the niosomes (Fig. 4A). Regarding the viability of biofilm-adhered cells, the cell count (CFU/mL) was considerably (*P* < 0.05) reduced by 55.66–61.5% after a 24-h treatment with STX solution. Encapsulation of STX within niosoma nanovesicles significantly enhanced efficacy and reduced the biofilm viability by 68–93% (Fig. 4B). Furthermore, the notable decrease in mature biofilms was investigated and illustrated using a bright field microscope, as depicted in (Fig. 4C).

**Fig. 5:**
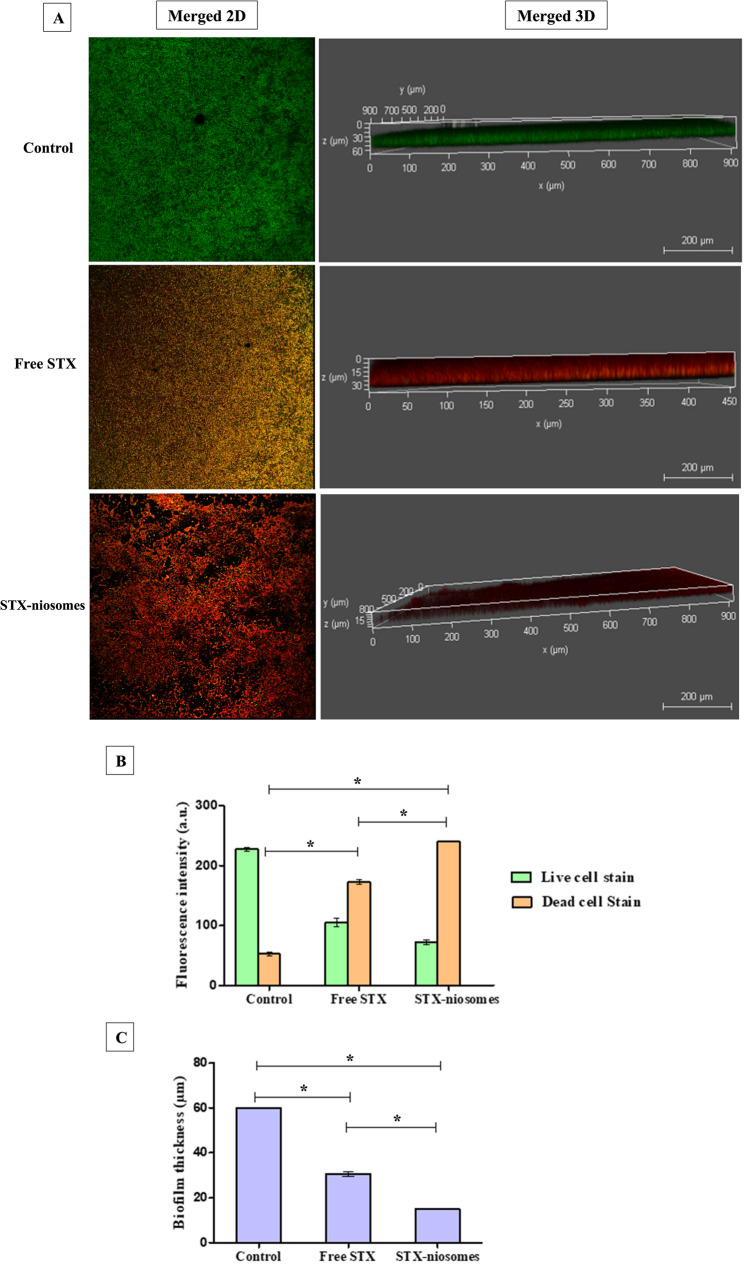
CLSM examination of the biofilm viability and thickness after and before STX encapsulation. (**A**) Representative images demonstrating the LIVE/DEAD-stained cells and 3D biofilm thickness with and without treatment. (**B**) Bar chart indicates fluorescence intensity represented in arbitrary units. (**C**) Bar chart indicates the 3D analysis of biofilm biomass thickness. Statistical significance is denoted by the asterisks, at *P <* 0.05 (one-way ANOVA). Three distinct areas of the preformed biofilm were used to measure the fluorescence intensity and biofilm thickness (z-stack)

The analysis of biofilms was further conducted through confocal microscopy to deepen the comprehension of the influence of STX (free and niosomes) on the structural elements of the biofilm. The fluorescence intensity indicated the percentage of LIVE/DEAD-stained cells with the biofilm matrix. Following 24 h of treatment, there was a notable decline in the viability of biofilm-embedded cells, with reductions of 56.8% (STX solution) and 79.1% (niosomes), as evidenced by the pronounced intensity of red fluorescence observed through propidium iodide staining (Fig. 5). Moreover, 3D-dimensional analysis indicated that the 48-h pre-established biofilm displayed a well-organized and substantial biofilm structure. Treatment administration for 24 h disturbed the preformed biofilm and significantly reduced (*P* < 0.05) the biofilm’s thickness by 50% (STX solution) and 75% (niosomes), as shown in Fig. 5.

.

### Virulence attenuation after staphyloxanthin treatment

**Fig. 6:**
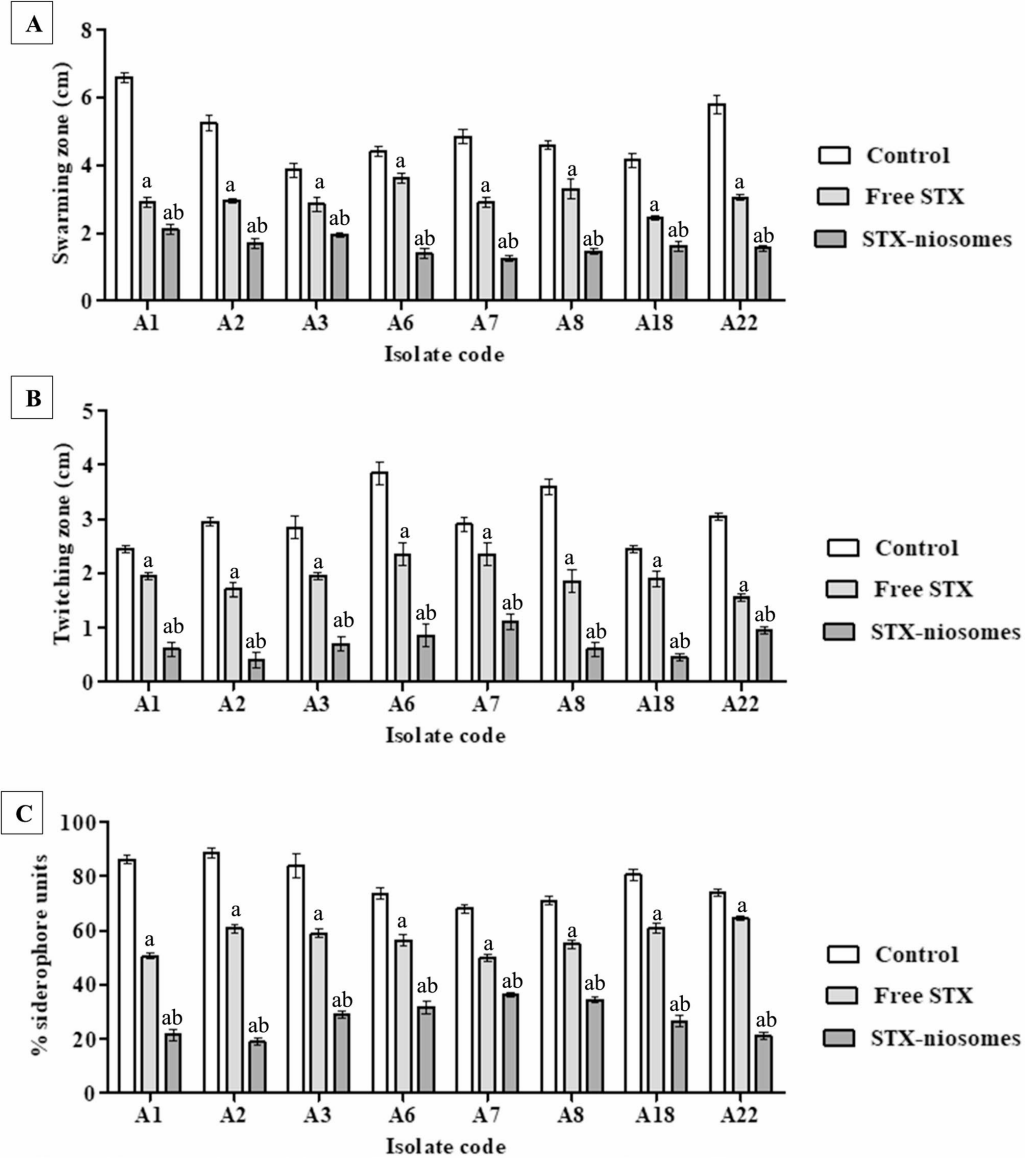
Attenuation of the virulence factors in *A. baumannii* isolates following treatment. (**A**) Bar chart indicates the reduction percentage in swarming motility. (**B**) Bar chart indicates the reduction percentage in twitching motility. (**C**) Bar chart indicates the reduction percentage in siderophore units. Statistical significance is denoted by the letters, at *P <* 0.05 (one-way ANOVA). Letter a indicates statistical significance compared to control. Letter b indicates statistical significance compared to treatment (free STX). Data expressed as mean ± SD (*n* = 3)

The impact of STX (free and niosomes) on the virulence determinants of *A. baumannii* isolates, including surface-associated motility, twitching motility, and iron acquisition activity, was evaluated. Using a semi-solid medium (0.5% MHA), an anti-surface motility assessment was carried out. The bacterial cells were treated with the STX solution at a sub-growth-inhibitory concentration (1/2 MIC), resulting in a notable decrease in surface motility of up to 47.29–63.07% (*P* < 0.05). The encasement within niosomal nanovesicles demonstrated markedly improved performance compared to the free solution, exhibiting a reduction in surface motility of up to 66.66 to 94.45% (Fig. 6; supplementary material S4). The twitching motility was evaluated utilizing a semi-solid medium (1% MHA). Following crystal violet staining, the dispersion from the inoculation site (toothpick stabbing) was notably reduced (*P* < 0.05) by 27.02–56.75% with the application of STX solution. The results illustrated in Fig. 6B indicated that the boosted diminution of dispersion (72.97–91.89%) is associated with the administration of niosomes.

It is worth noting that *A. baumannii* is able to adjust to an iron deficiency by producing and secreting siderophores, which are iron chelators. Therefore, a sub-growth-inhibitory concentration (1/2 MIC) of STX was evaluated for siderophore inhibition. After the addition of the CAS-HDTMA solution, the untreated cells exhibited a notable production of siderophores, as evidenced by the recorded OD values and the appearance of a pinkish-red color, a hallmark for the production of hydroxamate-type siderophores (supplementary material S4). The results displayed in Fig. 6C revealed that the percentage of siderophore units was significantly reduced (*P* < 0.05) after treatment by 26.68–44.06% (STX solution) and 48.75–79.5% (niosomes).

### Impact of staphyloxanthin on quorum sensing attentuation

**Fig. 7:**
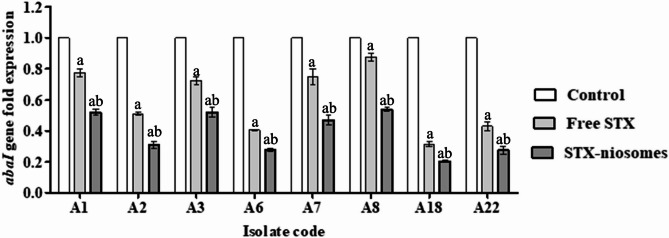
The impact of STX treatments (free and niosomes) on gene expression level of *abaI*. Statistical significance is denoted by the letters, at *P <* 0.05 (one-way ANOVA). Letter a indicates statistical significance compared to control. Letter b indicates statistical significance compared to treatment (free STX). Data expressed as mean ± SD (*n* = 3)

In *A. baumannii*, the expression of the *abaI* gene is a prerequisite for the two-component AbaI/AbaR quorum sensing system. Hence, qRT-PCR was employed to examine the effect of STX on the quorum sensing in the tested *A. baumannii* isolates by assessing the expression levels of the *abaI* gene after STX treatment at a concentration of 0.5 MIC. The results displayed in Fig. 7 indicated that the expression levels of *abaI* gene were significantly decreased (*P* < 0.05) by different extents in all examined isolates after STX treatment, with reduction percentages of 20.3%−68.5% (free STX) and 48%−79% (niosomes).

**Fig. 8:**
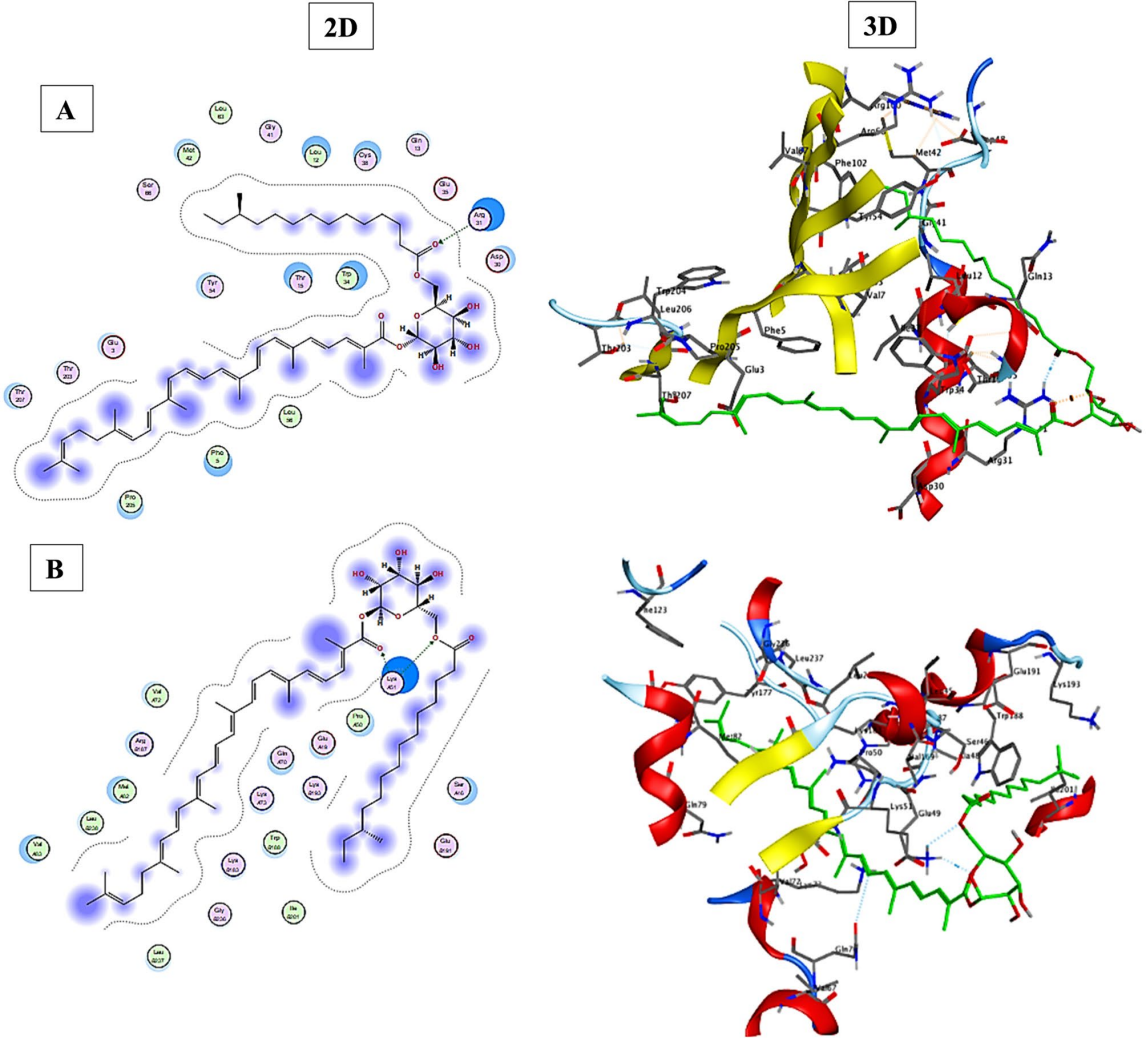
Molecular docking analysis demonstrating the docking and minimization of the STX residue in the binding pocket of quorum sensing proteins in both 2D and 3D. (**A**) AbaI (PDB code: 1KZF) and (**B**) AbaR (AlphaFold DB model; UniProtKB: A0A013UP)

The interaction between STX and the autoinducer synthase enzyme (AbaI protein) was subsequently examined to gain deeper insights into the plausible mechanism through which STX exerts its anti-quorum sensing activity. A molecular docking study was conducted using the “molecular operating environment (MOE) version 2019.0102” to elucidate the binding modes of the purified STX pigment with the the crystal structure of AbaI protein (PDB code: 1KZF). The results indicated that STX exhibited a significant docking score (−9.4302 kcal/mol) and was appropriately positioned within the active site of the AbaI protein. STX established a strong complex with the AgrA protein’s through hydrogen bonding interaction with Arg31 amino acid, as depicted in Fig. 8. The docking analysis was elaborated upon to explore the binding modalities between STX and AbaR (AlphaFold DB model; UniProtKB: A0A013UP), a receptor protein for N-acyl-homoserine lactones (AHL). The results illustrated in Fig. 8 indicated that STX was oriented effectively, forming a strong binding score (−8.9635 kcal/mol) through hydrogen bonding interaction with Lys51 within the active site of the AbaR protein. Ultimately, our findings suggested that STX may serve as a quorum sensing inhibitor through binding and interacting with the two-component AbaI/AbaR system, thereby inhibiting the expression of quorum sensing genes and other genes regulated by quorum sensing associated with virulence factors such as motility and biofilm formation.

### Staphyloxanthin nanovesicles effectively eradicate the meropenem-induced persistence

In the late exponential phase, bacterial cultures exhibited the formation of persister cells when confronted with meropenem stress at a concentration of 5X MIC for the tested *A. baumannii* isolates A1 (MIC = 8 µg/mL), A2 (MIC = 8 µg/mL), A7 (MIC = 16 µg/mL), and A8 (MIC = 16 µg/mL). After 24-h incubation, the time-dependent assay demonstrated that the maximum persister rates were 0.92% for isolate A3 and 0.58% for isolate A7, while the minimum levels were 0.089% for isolate A1 and 0.008% for isolate A8. This pattern was characterized by a typical biphasic killing curve, showcasing an abrupt drop in vulnerable cells followed by a plateau representing the subpopulation of the surviving persisters. The inflection points of the plot were recorded following a 4-h exposure to 5X MIC of meropenem (supplementary material Fig. S5). The culture derived from persister cells exhibited the same sensitivity to meropenem as the parent culture, with no observed change in MIC.

**Fig. 9:**
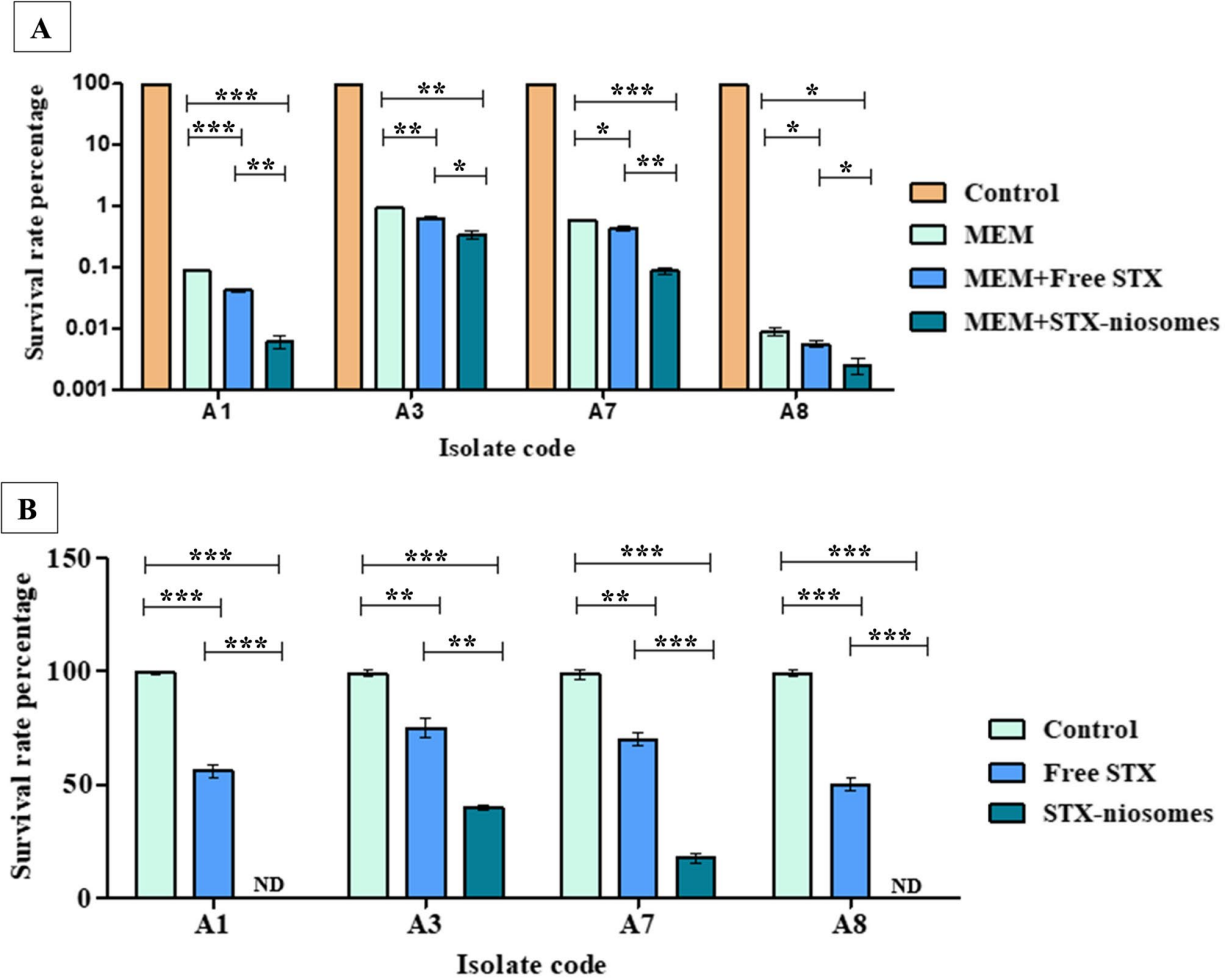
Effect of STX treatments on meropenem persistence against late exponential phase cells of *A. baumannii* isolates. (**A**) Survival rates of persister cells formation after exposure of meropenem (5X MIC) in conjunction with STX treatments for 6 h. (**B**) Survival rates of the pre-formed persister cells after treatments with STX (free and niosomes) for 6 h (ND = not detected). Statistical significance is denoted by the asterisks, at * *P <* 0.05, ** *P <* 0.01, *** *P <* 0.001 (one-way ANOVA). Data expressed as mean ± SD (*n* = 3)

The impact of meropenem exposure in conjunction with STX treatments (both free and niosomal) on persister formation was examined. Upon exposure of the cells in the late exponential phase to free STX, the formation of persister cells was significantly reduced by 28.4 ± 2.1% (A7), 32.4 ± 2.1% (A3), 44.44 ± 1% (A8), and 51.72 ± 3% (A1). The results displyed in Fig. 9A indicated that he combination of the niosomal formulation of STX with 5X meropenem was the most effective (*P* < 0.05) in reducing the survival rates of *A. baumannii* persistence compared to the free form of STX. In addition, the persister cells that survived were collected after 6 h, which was adequate for obtaining persister cells according to time-dependent assays, and were subsequently tested for STX eradication. The death rate of the pre-formed persister cells was accelerated after STX treatment by 23.5–48.2% in the all examined *A. baumannii* isolates. Intriguingly, no persister cells were detected when STX niosomal nanovesicle was applied in case of *A. baumannii* A1 and A8, as shown in Fig. 9B.

### In vivo assessment of the therapeutic efficacy of staphyloxanthin formulation

**Fig. 10:**
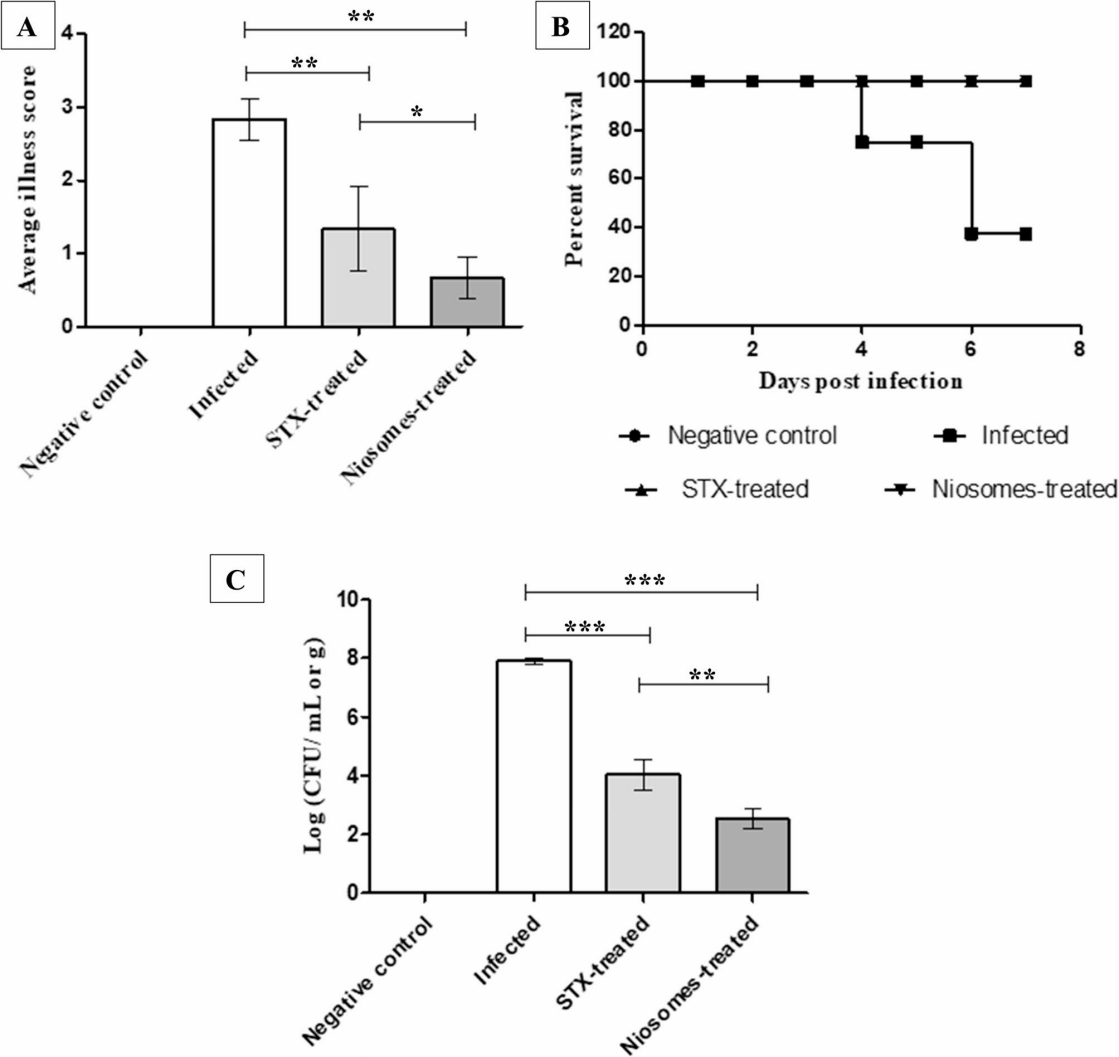
In vivo therapeutic efficacy of STX (free and niosomes) against respiratory infection of *A. baumannii* A8. (**A**) Bar chart demonstrating the average illness score during the infection study. (**B**) Kaplan-Meier survival curve demonstrating the mortality rate of mice before and after treatment. (**C**) Bar chart demonstrating the bacterial burden in lung homogenates of mice before and after treatment. Statistical significance is denoted by the asterisks, at * *P <* 0.05, ** *P <* 0.01, *** *P <* 0.001 (one-way ANOVA). Data expressed as mean ± SD (*n* = 3)

In the current study, we established a respiratory infection model to evaluate the therapeutic efficacy of STX (free and niosomes) in vivo. Consequently, we challenged different groups of neutropenic mice with *A. baumannii* A8 isolate (final inoculum size of 10^6^ CFU/mouse). The groups that received treatment (both free STX and niosomes) exhibited a markedly enhanced resistance (*p* < 0.05) to *A. baumannii* A8 respiratory infection, as evidenced by the clinical manifestations observed in the mice. Throughout the period of the observation, the animals administered the free-STX treatment displayed stage 2 clinical manifestations, as evidenced by the findings illustrated in Fig. 10A (moderate sensitivity to auditory stimuli and alterations in motility, a paler appearance than previously noted, along with a slight reduction in weight). Fortunately, there was a significant improvement in clinical symptoms (reduced weight loss and normal ear color, *p* < 0.05) in the group that received the niosomal formulation of STX, as evaluated by a lower clinical signs score (> 1). In contrast, the respiratory infection induced by *A. baumannii* A8 resulted in a marked deterioration of the clinical signs noted in the untreated group, culminating in an exacerbation of the disease progression to a deadly outcome (stage 3, characterized by cyanosis and mortality; Fig. 10A).

**Fig. 11:**
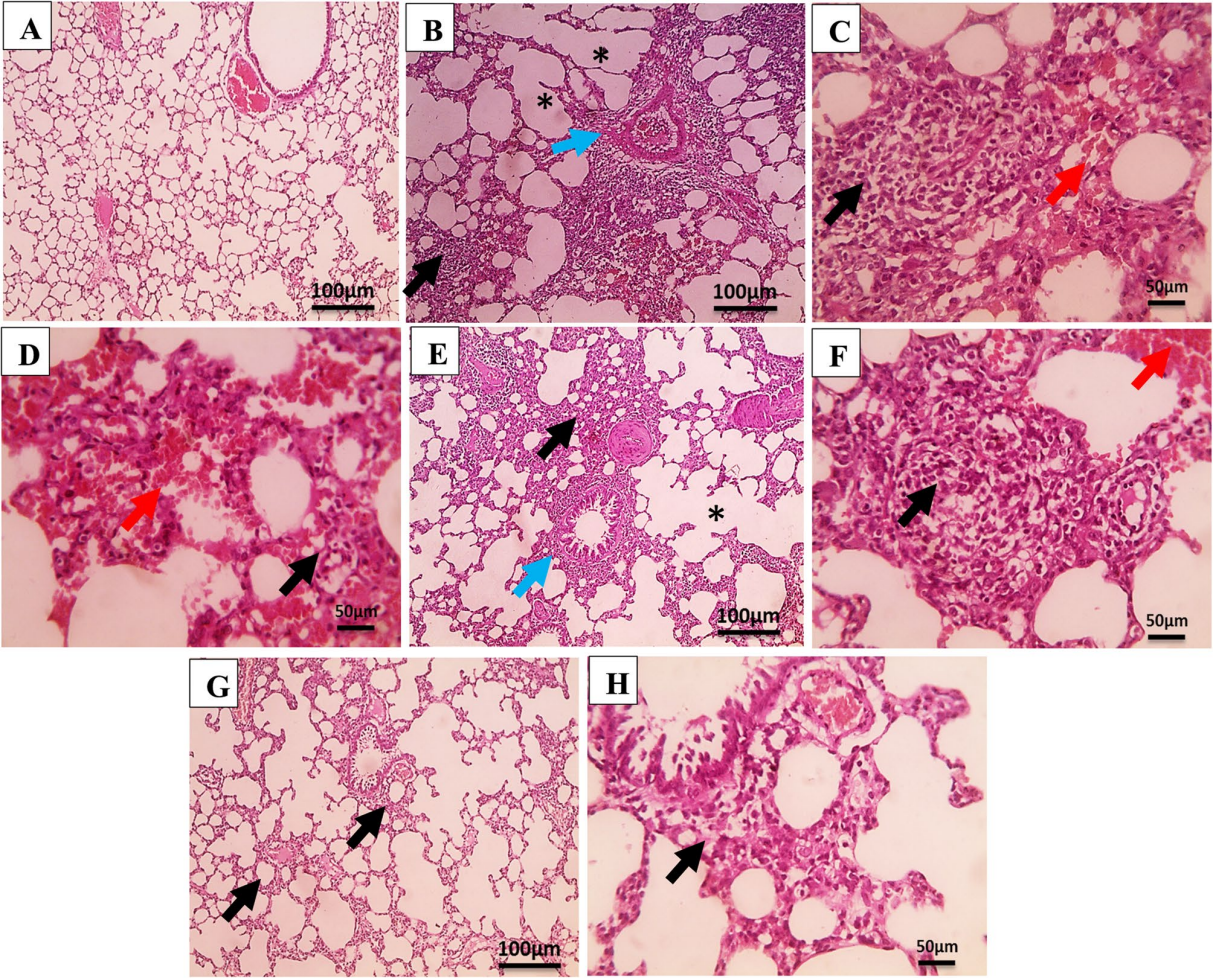
Histopathological findings of H&E-stained lung tissue sections of infected mice and treated with different treatments after 6 h of *A. baumannii* A8 inoculation. (**A**; 100x) photomicrographs of lungs sections from normal control group (GI) showing no structural damage and inflammation with normal bronchiole surrounded by normal sized alveoli. (**B**; 100x), (**C**; 400x) and (**D**; 400x) photomicrographs of lungs sections from infected untreated group (GII) showing interstitial inflammation (black arrows) and perivascular inflammation (blue arrow) separated by congested alveoli (red arrows) with areas of destructed emphysematous alveoli (*). (**E**; 100x) and (**F**; 400x) photomicrographs of STX-treated group (GIII) lungs sections showing multifocal interstitial inflammation mainly macrophages (black arrows), mild peribronchial inflammation (blue arrow), areas of mild congestion (red arrow) and mild alveolar emphysema (*). (**G**; 100x) and (**H**; 400x) photomicrographs of niosomes-treated group (GIV) lungs sections showing weak interstitial inflammation (black arrows)

The results from the Kaplan-Meier survival curve (Fig. 11B) corroborated these observations. At the conclusion of the seven-day monitoring period, the treatment with STX (free and niosomes) had successfully rescued the entire treated mice. The survival rate of the untreated group, in contrast, was markedly diminished at 80% by the day four of the experiment, and this decline persisted, ultimately reaching 40% by days six and seven (Fig. 10B). The enhanced clinical outcomes and boosted survival rates stated substantiate the curative potential of STX in vivo in combating the induced lethal infection of *A. baumannii*. The killing capacity of STX was further evaluated through an evaluation of the bacterial load in lung homogenates. In the untreated group, the lung samples showed a bacterial burden of 7.9 ± 0.1 (mean log (CFU/g)) after 48 h of inoculation. Mice receiving STX solution showed a significant reduction (*p* < 0.05) of bacterial counts to 4.027 ± 0.5021 log units. The best improvement in lowering bacterial load (*p* < 0.05) was observed in case of administration of niosomal nanovesicles (2.533 ± 0.3215 log units), as shown in Fig. 10C.

### Niosomal formulation of staphyloxanthin alleviates pulmonary destruction and inflammation triggered by lung infection

The efficacy of STX (free and niosomes) in protecting the lungs from a deadly respiratory infection was assessed by histologically examining the lung tissues to identify pathological alterations. As expected, the normal control’s H&E-stained lung tissues showed no structural damage or inflammation, along with appropriately sized alveoli and bronchioles (Fig. 11A). The untreated infected group displayed patches of destructed emphysematous alveoli and extensive congested surrounding blood vessels, which were clear signs of inflammation. There was extensive bronchial and interstitial inflammation in the pulmonary alveoli. Furthermore, inflammatory cell infiltration was observed, primarily consisting of macrophages with a limited count of neutrophils (Fig. 11B-D). The infected group treated with STX solution exhibited histopathological findings of multifocal interstitial inflammation primarily consisting of macrophages, alongside mild peribronchial inflammation, characterized by spots of mild congestion and alveolar emphysema (Fig. 11E&F). Encapsulation of STX within nanovesicles, on the other hand, showed matured granulation tissue that was well-formed, enriched with freshly generated capillaries, and showed elevated fibroblast recruitment. Additionally, lung tissue displayed moderate fibrosis, an average-sized bronchiole with minimal inflammation, and alveoli of average size connected by thick fibrous septa (Fig. 11G&H).

Semi-quantitative histological metrics ranging from minimal-to-widespread appearance were also used to evaluate the effectiveness of STX and indicate the severity of the pathological findings. It was demonstrated that mice treated with STX formulation exhibited improved histology scores over the untreated group (GI) and the group treated with unencapsulated STX. Results in Table 5 unequivocally indicated that STX treatment reduced the bacterial infection-induced congestion and degradation of the alveoli, as well as the inflammation of the interstitial and bronchial tissues in the lungs.

**Table 5:** Semi-quantitative assessment of histological parameters

Histological parameters	Infected group	STX-treated group	Niosomes-treated group
Alveolar congestion	++++	+	-
Inflammation	++++	+++	+
Fibroblasts proliferation (fibrosis)	+	++	++
Emphysema	+++	++	+
Angiogenesis	+	++	++
Granulation tissue	+	++	+++
Collagen deposition	-	-	-

Absence -, Mild +, Moderate ++, Severe +++, Widespread ++++

## Discussion

*A. baumannii* is an opportunistic pathogen of considerable healthcare magnitude, held accountable for various resilient nosocomial infections globally, especially in patients with serious illnesses [1]. Multidrug-resistant *A. baumannii* is a highly concerning bacterium that conveys noteworthy health threats owing to the scarcity of effective treatment options [44]. Consequently, exploring innovative strategies for their management is a necessity for minimizing elevated mortality rates stemming from therapeutic inadequacies. The astounding biological and antimicrobial attributes of natural pigments such as carotenoids and apocarotenoids render them fascinating candidates [17]. However, the potential efficacy of carotenoids may be hindered by poor solubility and bioavailability [45]. To overcome this challenge, a novel approach through the delivery of STX in the form of nanovesicles was formulated for augmenting the therapeutic efficacy as a promising antibacterial agent [14, 45].

This was accomplished through the encapsulation of STX within niosomes for enhanced therapeutic efficacy in combating *A. baumannii* planktonic cells and biofilms. Niosomes, which are vesicular carriers composed of cholesterol and non-ionic surfactants, encapsulated STX, thereby facilitating its attainment of the intended effect. The encapsulation of hydrophilic and lipophilic medications utilizing niosomes is indeed achievable [46]. The intriguing possibility of niosomes to enhance biological activity in the realm of pathogen defense warrants deeper exploration [16]. It was suggested that these carriers could improve the drug’s ability to penetrate the pathogen membrane, demonstrate an enhanced antibacterial effect, and minimize its detrimental impact on healthy cells [47, 48]. The data provided strong support for our decision to utilize niosomes as the delivery mechanism. This study primarily involved the administration of cholesterol through niosomes produced with span 60 serving as the main surfactant [49]. Drug retention and entrapment may be enhanced by adding cholesterol as a stabilizing factor to the membrane. The prepared vesicles displayed a spherical morphology, as expected. The measured dimensions of the vesicles varied between 100 and 200 nm within the nanoscale range. The reported data exhibits a consensus regarding both morphology and size values [50, 51]. It is essential to emphasize that the properties of the drug and the particular category of lipid are fundamental factors influencing the efficacy of drug entrapment, especially concerning lipophilic drugs [23]. The recorded drug loading and entrapment efficiency is underscored by evidence indicating that STX, due to its lipophilic nature, predominantly resides within the lipid core of the vesicle. The observed entrapment efficiency and release profile align with the values reported by other researchers, indicating they fall within the appropriate range [15, 52, 53].

This report represents, to our knowledge, the first examination of the impact of STX in the fight against *A. baumannii*. This involved a thorough analysis of the effects of free and niosomes-encapsulated STX on planktonic cells and biofilms. Our study indicates that STX exhibited significant antibacterial activity against all tested *A. baumannii* isolates, as evidenced by the recorded MIC values (Table 4). The notable antibacterial efficacy of STX can be fundamentally attributed to its inherent chemical composition of carotenoids. In summary, the biological potential of carotenoids is largely linked to their role in generating reactive oxygen species, which subsequently leads to oxidative stress and modifications in cell permeability [10, 54]. One plausible mechanism by which pro-oxidative features increase antibacterial potential was proposed by Black et al. [55]. This mechanism entails disrupting with the pathogens’ ability to generate energy, bind to their membrane glycoprotein, and decouple oxidative phosphorylation [55]. Moreover, the antibacterial activity was increased by niosomal encapsulation of STX, as evidenced by a lower MIC when compared to the comparable STX solution (Table 4). The tendency of vesicular systems to adsorb to the bacterial cell membrane has been the central theme of numerous studies elucidating the mechanism behind enhanced antibacterial efficacy following encapsulation within niosomes as vesicular carriers [16]. Fusion of vesicles to the bacterial surface was proposed in other reports [56, 57]. The adsorption or fusion process can render the bacterial cell wall more permeable, facilitating an increased influx of medication into the bacterial cells. Even in biofilm-forming bacteria, it is thought that these effects reduce bacterial resistance [57, 58]. The documented findings observed across various bacterial strains are consistent with the magnitude of enhancement in antibacterial activity complying with niosomal encapsulation [15, 16, 59]. Consistent with our research, the co-delivery of curcuminrosemary loaded niosomes showed higher antimicrobial activity with lower MIC values and successfully inhibited the growth of the pathogenic bacteria [60]. Another recent study reported that niosomes encapsulating carvacrol displayed up to 4-fold higher antibacterial activity against methicillin-resistant *Staphylococcus aureus*, compared to free drug [61].

In addition to its effects on planktonic cells, STX demonstrated notable efficacy in inhibiting biofilm formation as well as disrupting pre-established biofilms. Biofilm production is regarded as one of the most critical virulence factors in *A. baumannii* [62]. The process of biofilm formation commences with the adhesion of microbes to abiotic and biotic surfaces, such as endotracheal tubes and medically relevant surfaces. After the initial adhesion, bacteria will proliferate and aggregate to form microcolonies. The completely formed biofilm undergoes maturation and maintenance [28, 62]. This investigation revealed that STX successfully inhibited biofilm formation at multiple stages, including initial adhesion (cell surface hydrophobicity), biofilm matrix (exopolysaccharide and crystal violet assay), and mature biofilm (light and confocal microscopy), especially in the context of niosomal encapsulation. The encouraging potential of niosome-encapsulated natural products in the management of biofilm-forming bacteria has been demonstrated in a multitude of research studies [63, 64]. Our study aligns with recent research reporting the potential efficacy of β-carotene extracted from *Azanza garckeana* in combating respiratory infections caused by *A.* baumannii and other ESKAPE pathogens by inhibiting biofilm formation and eradicating mature biofilms [65].

An intriguing approach for attenuating the pathogenicity of *A. baumannii* involves targeting the quorum sensing network. The two-component AbaI/AbaR system, a homologue of the LuxI/LuxR system seen in other Gram-negative bacteria, comprises up *A. baumannii*’s quorum sensing system [66]. The enzyme autoinducer synthase, known as AbaI, is encoded by the *abaI* gene, which plays a crucial role in the synthesis of N-acyl-homoserine lactone (AHL). With the accumulation of AHL, the receptor protein AbaR, encoded by *abaR*, initiates a series of reactions that subsequently modify the expression of various genes regulated by quorum sensing. This includes genes associated with virulence factors such as biofilm formation, motility, and iron acquisition [67]. Blocking the expression of the autoinducer synthase encoding gene (*abaI*) leads to quorum quenching and downregulation of virulence genes [66, 67]. Our investigation revealed that STX effectively suppressed the expression of the *abaI* gene across all examined *A. baumannii* isolates. The quorum quenching activity of STX has been proven through an observed attenuation in bacterial motility and siderophore production at sub-inhibitory concentrations, preserving the viability. Additionally, the computational analysis revealed the anti-quorum sensing activity of STX through binding with the two-component proteins (AbaI and AbaR) of the quorum sensing system. Consistent with our research, the ethanolic extract of *Azadirachta indica* showed its potent quorum quenching activity by downregulating the expression of *abaI* gene, which subsequently leading to a decrease in the motility and iron acquisition of *A. baumannii* [68]. Additionally, Pumirat et al. observed that *abaI* mutations showed notable changes in protein expression, particularly in proteins associated with virulence, antibiotic resistance, and membrane structure, between mutant and wild-type strains [69].

Nevertheless, high persister emergence rates in *A. baumannii* under antibiotic stress have been documented [8, 39, 40]. A subpopulation of bacteria termed persister cells demonstrates resilience to antibiotic treatment, potentially leading to therapeutic failure and recurrence, particularly in recalcitrance of chronic infections, upon cessation of antibiotic pressure. The eradication of persisters is crucial for managing antibiotic tolerance and for developing new strategies to enhance treatment efficacy and reduce patient stress [70]. This study demonstrates that the encapsulation of STX within niosomal nanovesicles significantly inhibited the formation of persisters in the presence of meropenem and effectively eradicated the pre-formed persistence. Our study aligns with previous research indicating that thymol was utilized either alone or in conjunction with meropenem for anti-persister activity against *A. baumannii* [8]. Another instance is curcumin, which reinstated the sensitivity of colistin-persisted *A. baumannii* owing to its pro-oxidant properties [39].

To further confirm the efficacy of STX against *A. baumannii* infection, we evaluated the impact of STX on respiratory infections induced by antibiotic-resistant *A. baumannii* isolates, which are frequently identified in immunocompromised patients with pneumonia [42, 71]. Upon elaboration, we observed a statistically significant improvement in clinical outcomes among the treated groups, evidenced by higher survival rates, reduced bacterial burden, and decreased inflammation in lung tissues. Our study aligns with another report indicating that intranasal administration of *Scutellaria barbata* extract resulted in 100% survival of infected mice with *A. baumannii* and demonstrated improved resolution of alveolar, peribronchial, and perivascular inflammation [72]. However, to date, there is no published data reporting the antivirulence and antibiofilm potentials of STX in either drug solution or niosomal dispersion.

## Conclusion

Encapsulation of STX in niosomal nanovesicles presents a novel anti-virulence strategy against multidrug-resistant *A. baumannii*, effectively disrupting quorum sensing and the survival of persister cells. The in vivo efficacy underscores clinical promise; however, comprehensive safety studies, delivery optimization, and validation in advanced preclinical models are required before translation into clinical application.

## Supplementary Information

Supplementary Material 1.

## Data Availability

All the data supporting the research results are available in this the article.
