# Supplementary material for: Deciphering the efficacy of staphyloxanthin-encapsulated niosomal nanovesicles to attenuate biofilm formation, quorum sensing, and meropenem persistence in Acinetobacter baumannii
Source: BMC Microbiol. 2025 Dec 13;25:791. doi: 10.1186/s12866-025-04507-1 (PMC12701606; doi:10.1186/s12866-025-04507-1)
Supplement: Supplementary file 1 — Supplementary Material 1. [file 12866_2025_4507_MOESM1_ESM.docx]

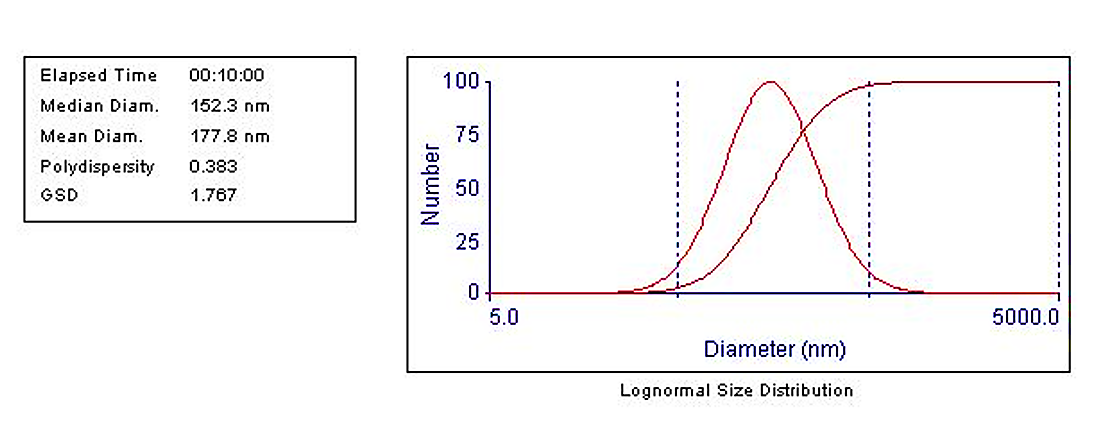


**A**

**B**


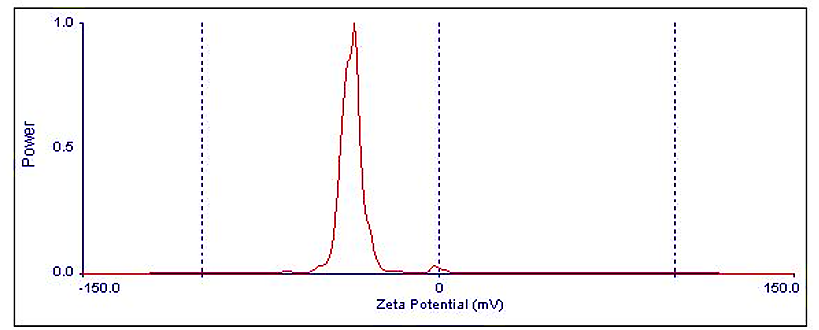


**Figure S1** Characterization of the formulated niosomes. (A) DLS analysis of niosomes size and distribution. (B) zeta potential measurement of niosomes.

**A**


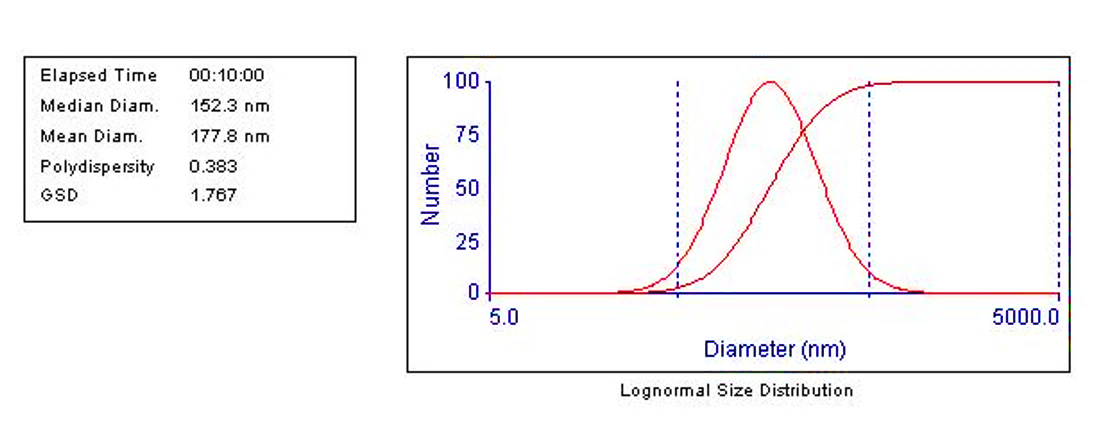


**B**


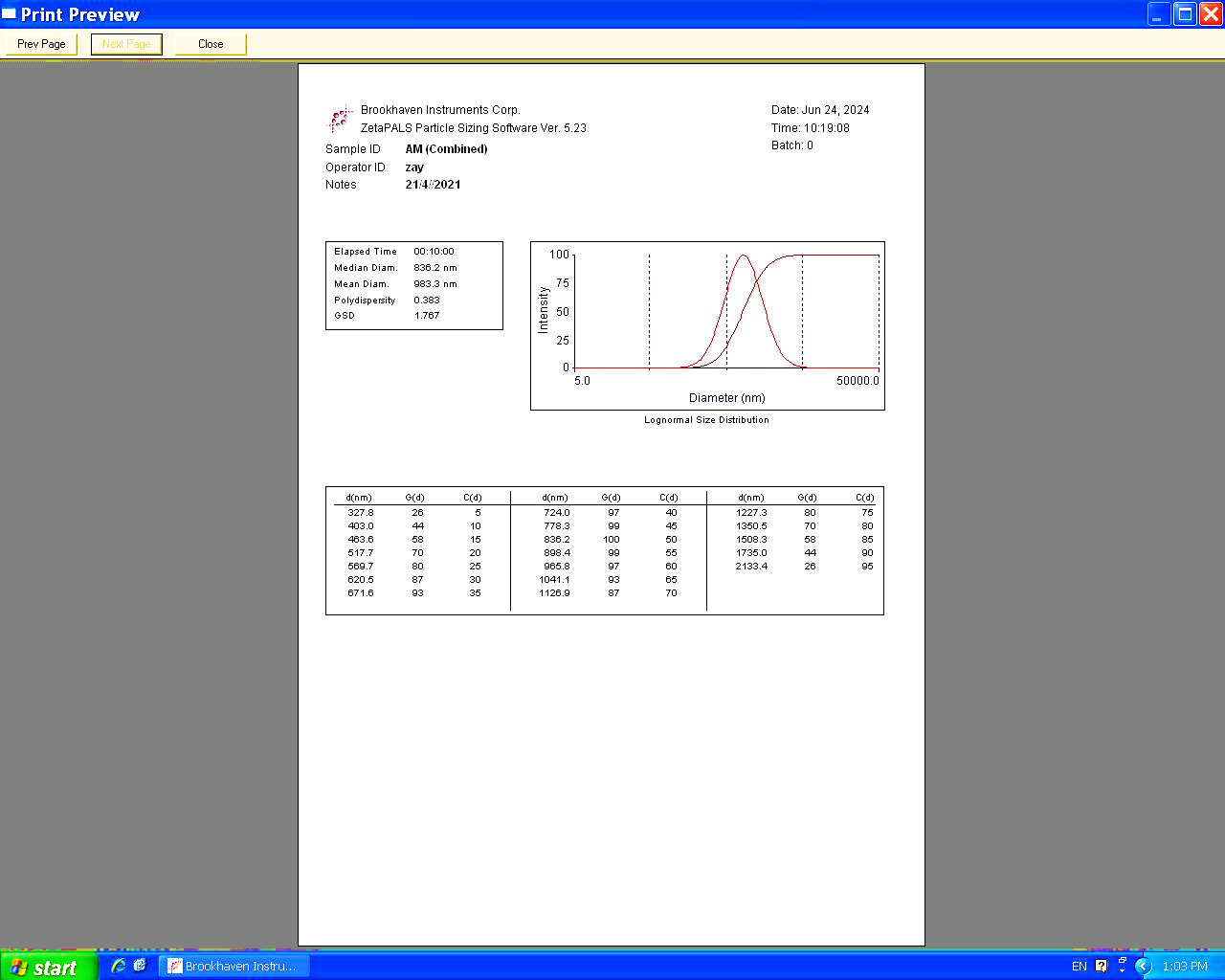


**Figure S2** Stability of the formulated niosomes over a 30-day period at different temperatures of 4 °C and 25 °C. (**A**) DLS analysis showed niosomal size in the nanoscale range in case of refrigeration. (**B**) DLS analysis showed aggregation of nanovesicle after storge at room temperature.


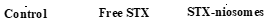

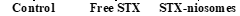

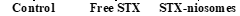

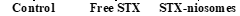

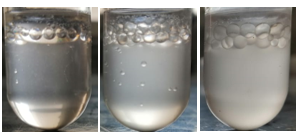


**A**

**B**


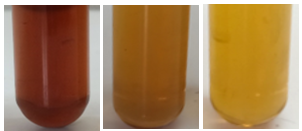


**C**


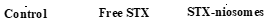
**
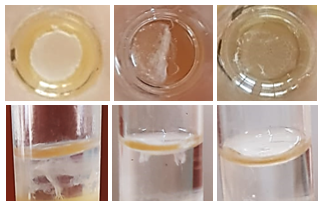
**

**D**


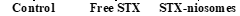

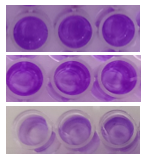


**Top view**


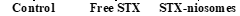


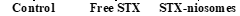


**Side view**


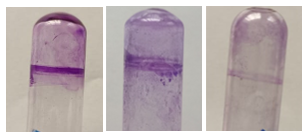

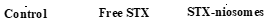


**E**

**Figure S3** Representative images demonstrating the effect on cellular adhesion and biofilm formation of strong biofilm producing *A. baumannii* isolates. (**A**) The affinity of cells to the non-polar solvent (toluene) with and without treatment. **(B)** The colored supernatants produced by phenol–sulfuric acid method. (**C**) The intensity of crystal violet staining with and without treatment. (**D**) The formed pellicles with and without treatment. (**E**) Representative images demonstrating the ring biofilm formation with and without treatment.


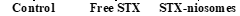

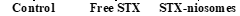

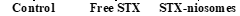


**A**


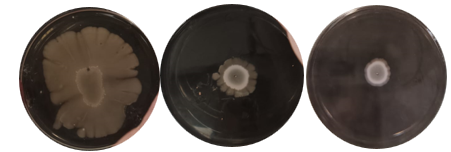


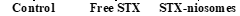


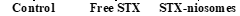

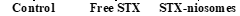


**B**


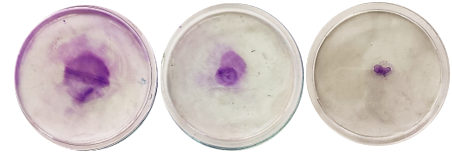


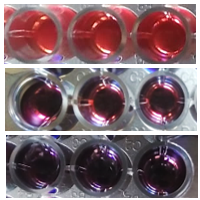


**C**


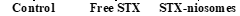


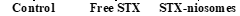


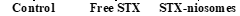


**Figure S4** Representative images demonstrating the attenuation of the virulence factors in *A. baumannii* isolates following treatment. (**A**) zones of swarming. (**B**) zones of twitching. (**E**) The color of wells after addition of CAS-HDTMA solution due to reduction of sideropjore production.

**
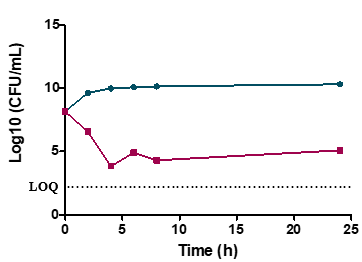
**
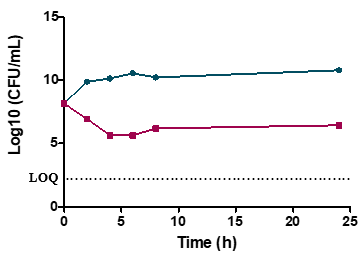

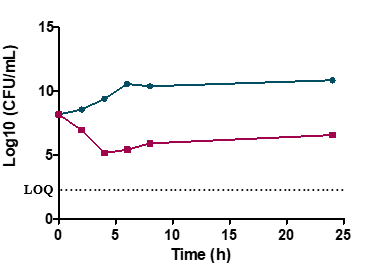


**D**

**C**

**B**

**A**


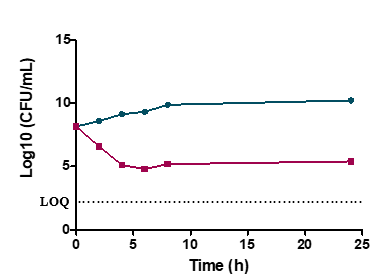


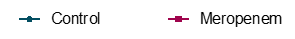


**Figure S5** Biphasic kill curves of *A. baumannii* isolates A1 (**A**), A3 (**B**), A7 (**C**), and A8 (**D**) under meropenem stress at a concentration of 5X MIC. Limit of quantification (LOQ) = 10^2.3^_._ Data represented as mean ± SD.
